# Rethinking chemical clusters: Greenfield and brownfield transition to net-zero by 2050

**DOI:** 10.1016/j.isci.2026.117077

**Published:** 2026-09-05

**Authors:** Julia L. Tiggeloven, André P.C. Faaij, Gert Jan Kramer, Matteo Gazzani

**Affiliations:** 1 Utrecht University, Copernicus Institute of Sustainable Development, Princetonlaan 8a, 3584 CB Utrecht, the Netherlands; 2 TNO, Energy Transition Studies, P.O. Box 37154, 1030 CE Amsterdam, the Netherlands; 3 Sustainable Process Engineering Chemical Engineering and Chemistry, Eindhoven University of Technology, 5612 AP Eindhoven, the Netherlands

**Keywords:** chemical clusters, mixed integer linear programming, MILP, net-zero emissions, scope 1–3 emissions, multi-period optimization, brownfield transformation, transition pathways

## Abstract

We develop a cluster-level optimization framework to design brownfield transition pathways toward net-zero chemical clusters by 2050, applied to existing ammonia-olefin clusters in the Netherlands. The model represents sequential investment decisions for 2030, 2040, and 2050, includes scope 1–3 emissions, and enables a systematic comparison of greenfield and brownfield configurations. Thirteen olefin and eight ammonia production routes are considered. The results show that, despite reduced flexibility due to legacy assets, net-zero brownfield configurations remain feasible. Plastic gasification combined with methanol-to-olefins emerges as a low-regret option for olefins, while electric steam methane reforming and electrolyzers are robust choices for ammonia. The results support early investment rather than strategic delay. Excluding scope 3 emissions leads to lock-in of fossil-based options that appear cost-effective short-term but raise long-term system costs. Sensitivity analyses highlight uncertainties related to infrastructure and technology availability, bio-feedstock prices, and plastic waste emission accounting.

## Highlights

• Brownfield chemical clusters can reach net-zero by 2050
• Low-regret routes: plastic gasification, CO_2_ electrolysis, MTO, electrified SMR
• Early investment can reduce long-term costs and avoids fossil lock-in
• Scope 3 emissions and infrastructure critically shape transition pathways

## Graphical abstract

**Figure:**
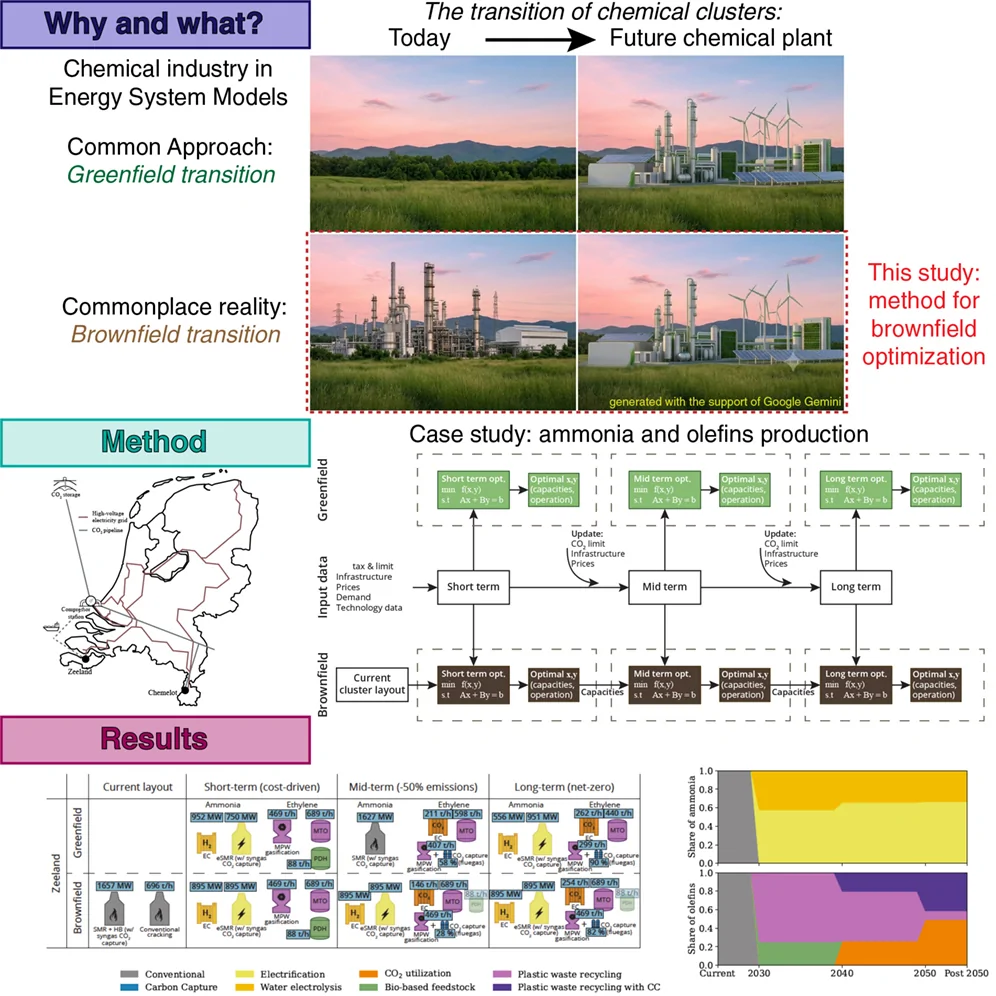

## Teaser

Environmental science; Global change

## Introduction

The chemical industry plays an essential role in modern society, forming the backbone of countless products that define our daily lives. Transitioning this industry toward more sustainable practices, addressing both local pollution and greenhouse gas emissions, is widely recognized as imperative. Yet, there is no clear consensus on the most effective pathways to achieve this transition. Several factors make this transition particularly challenging. A key obstacle is the carbon embedded in many chemical products, which extends the emissions footprint beyond the production phase, and which necessitates coordinated actions on scope 1, 2, and 3 emissions—a unique challenge compared with other sectors.^1^ Additionally, the industry has high energy consumption, with many processes requiring high temperatures and pressures. The combined requirement for energy supply and carbon feedstock makes fossil fuels particularly difficult to replace, yet this transition is unavoidable. The economic landscape further complicates the transition: the chemical industry often operates on thin profit margins per ton of product, requires significant capital investments, and depends on long-lasting infrastructure. In Europe, many existing facilities would need to be replaced to meet sustainability goals, while starting from scratch in other regions might be more convenient. At the same time, existing facilities may still hold value or have components that can be reused. Global competition adds further pressure to ensure that efforts to reduce emissions do not undermine industrial competitiveness. Thus, a viable path to a sustainable chemical sector must ensure climate goals are met while preserving economic feasibility. Overall, the works of Sovacool et al.,^2^ Janipour et al.,^3^ and Tickner et al.^4^ present the different challenges of such transition: they converge on the insight that the transition of the chemical industry is constrained less by technology availability than by institutional lock-in, decision-making problems, and misaligned economic and policy frameworks.

To navigate these complexities, explore strategies, and develop technologies, researchers have increasingly relied on modeling (e.g., Diéguez et al.,^5^ Zibunas et al.,^6^ Huo et al.,^7^ and Gabrielli et al.^8^). However, existing models vary in scope and assumptions, limiting comparability.^9^ Key gaps include limited integration of low-carbon technologies across the available mitigation options, lack of harmonized data and scenarios, and insufficient resolution at regional or site levels. The importance of research at the site level was further emphasized by Gabrielli et al.,^8^ who compared net-zero pathways on a country level based on energy, land, and water use but also highlighted the need for more site-specific assessments.

Cluster-level transitions introduce additional socio-technical complexity. First, the availability and suitability of critical infrastructure and renewable electricity can significantly impact the feasibility of mitigation strategies.^10^ Second, process integration plays a central role in cluster-scale modeling; a network analysis by Tan et al.^11^ highlighted the importance of interconnections within chemical clusters and showed how the adoption of alternative feedstocks can fundamentally reshape these dependencies. Third, detailed operational modeling of technologies, especially when relying on electricity or renewable potential, requires hourly resolution and introduces complex constraints with integer variables and time-step linkages, resulting in substantial computational complexity. Fourth, the inclusion of individual plant capacities and economies of scale necessitates integer decision variables, further complicating optimization. Finally, the valuation of existing assets influences investment decisions, particularly in regions with extensive legacy infrastructure, where choices between replacement and reuse must be carefully evaluated.

While national or global linear models provide a broad view of emissions reduction pathways, they often lack local detail. For example, Diéguez et al.^5^^,^^12^ optimize long-term investments and allow for capacity decommissioning, but their linear structure ignores economies of scale and assumes partial plant closures, an assumption not viable at the cluster scale. Satymov et al.^13^ investigate an integrated national energy-industry system, showing how sector coupling, electrification, biomass, and CO_2_ capture can enable cost-optimal carbon neutrality in Nordic conditions. However, the chemical sector is represented at a stylized, system level, without explicit modeling of individual process technologies or detailed unit-level constraints. Meanwhile, cluster-level models with multiyear time horizons often lack detailed operational modeling. Starreveld et al.,^14^ for instance, apply robust multiyear optimization to Dutch industrial clusters but do not model hourly process dynamics. The recent work of Thakker and Bakshi^15^ contributes a rigorous multi-period optimization framework for chemical industry transitions across cradle-to-cradle life cycle networks; by integrating technology readiness evolution, integrated assessment models, and policy instruments, it identifies cost-effective and robust transition roadmaps under deep uncertainty. However, the time resolution is also in this case yearly, therefore without hourly process dynamics. Furthermore, scope 3 emissions remain a challenge at regional or local levels, as they depend on complex, interconnected supply chains extending beyond local boundaries. In earlier work, we addressed aspects like infrastructure constraints, hourly operations, and carrier synergies.^10^ Overall, significant gaps remain, including the valuation of existing assets in brownfield scenarios, the intermediate steps required for long-term transitions, the integration of technologies and solutions capable of handling scope 3 emissions, the possibility of using low technology readiness level (TRL) technologies, and the availability of feedstock switching.

This paper aims to tackle these modeling gaps by developing a comprehensive modeling framework that enhances the analysis of net-zero transition pathways for the chemical industry at a cluster level. Specifically, we

• develop a mixed-integer linear programming (MILP) optimization framework^10^^,^^16^ that can model the near, medium, and long-term transition of an ammonia-ethylene cluster, incorporating bio-based and negative CO_2_ pathways, as well as advanced and emerging technologies.
• expand the time horizon to include long-term net-zero emission targets while maintaining an hourly resolution, solving sequential decade intervals to reflect real-world transitions and evolving information availability.
• compare brownfield and greenfield scenarios to assess their respective challenges, advantages, and risks of technology lock-in.
• integrate scope 1, 2, and 3 emissions, ensuring a comprehensive assessment of emissions reduction pathways for two existing ammonia-ethylene clusters in the Netherlands (Chemelot and Zeeland).

Thanks to these improvements and novel aspects, this study advances the state of the art in modeling chemical industry transitions in several ways. It bridges the gap between national-scale linear models and detailed process studies by introducing a cluster-level optimization framework that simultaneously captures long-term transition dynamics, hourly operational detail, and the constraints imposed by existing infrastructure. By explicitly distinguishing between brownfield and greenfield pathways, the framework provides new insights into the role of legacy assets, the risk of technology lock-in, and the feasibility of staged transitions toward net-zero targets. Moreover, the integration of scope 1, 2, and 3 emissions within a unified MILP formulation enables a more comprehensive assessment of mitigation strategies. Beyond the specific case studies, the proposed framework provides a transferable methodological approach that supports more realistic, policy-relevant analyses of industrial transitions, enhancing scientific understanding of how technological, temporal, and infrastructural constraints shape viable transition pathways.

The next section discusses the case studies, providing the context for the analysis. This is followed by the methodological framework, which details the optimization problem, the integration of brownfield optimization, and the consideration of scope 3 emissions. We then present the results, followed by a discussion of the findings and a summary of the key conclusions.

### Pathways to net-zero chemical production

**Figure 1:**
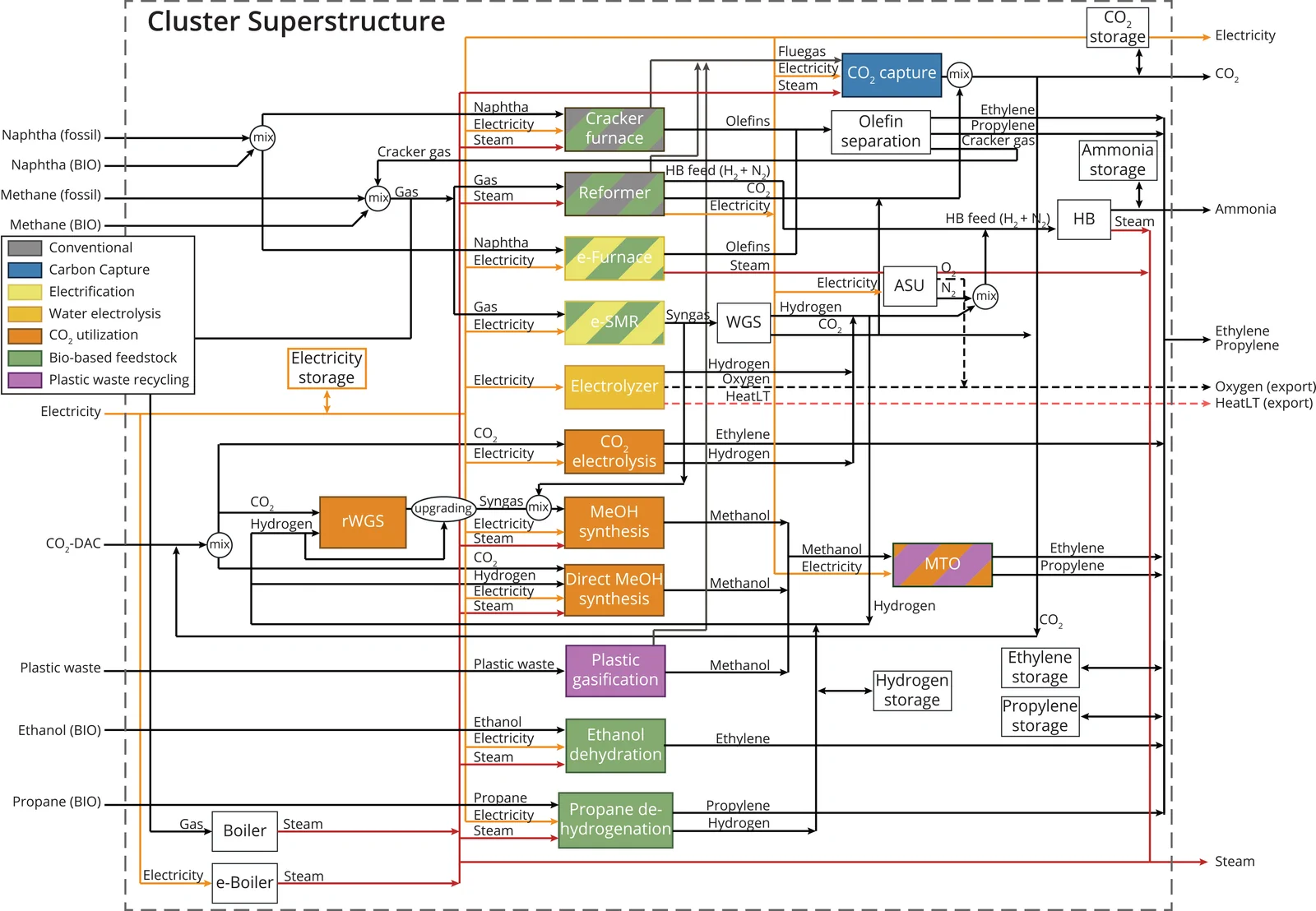
Overview of the production pathways considered in this work and their interconnections within the cluster superstructure The key processes are color-coded depending on the type of production pathway (e.g., yellow: electrification, orange: CO_2_ electrolysis, see legend), while the supporting equipment that can be used in all or several other pathways are left in white (e.g., storage).

In this work, we consider conventional and emerging production pathways for olefins and ammonia. Figure 1 shows the cluster superstructure, including all considered production routes along with associated auxiliary technologies and storage options. In total, the superstructure comprises 13 distinct olefin production routes and 8 ammonia production routes. The technologies considered in the superstructure cover all pathways identified as relevant for a net-zero chemical industry,^9^ while also keeping computation tractability viable. More specifically, we selected technologies that are either commercial or that have already reached the stage of relevant industrial demonstration; this decreases the uncertainty associated to technology implementation, as all routes—with the exception of CO_2_ electrolysis (discussed later)—are expected to be commercial within the time frame analyzed in the study.

### Conventional and (near-)commercial routes

Several chemical clusters rely on the same conventional processes for fertilizer and olefins production. Olefins production is based on the thermal cracking of naphtha, yielding light olefins, aromatics, hydrogen, and methane. The heat for the reactions is supplied by burning cracker gas—a by-product of the process composed of methane and approximately 11% hydrogen—in a pyrolysis furnace, thereby eliminating the need for additional chemical energy beyond naphtha. Conventional ammonia production follows the standard Kellogg Brown & Root (KBR) sequence of syngas production through primary steam methane reforming (SMR), secondary auto thermal reforming (ATR), and water gas shift (WGS), followed by ammonia synthesis via the Haber-Bosch (HB) process. While pure methane is typically used as both feedstock and fuel in ammonia production, this study also considers direct integration with olefin production through the utilization of cracker gas. As ammonia production is expected to remain dependent on the HB process, we analyze the reforming and synthesis stages separately, exploring sustainable alternatives for syngas production.

Both naphtha cracking and SMR involve high-temperature reactors or furnaces that rely on fuel combustion, leading to CO_2_ emissions. In naphtha cracking, all direct process emissions are released through the flue gas. For ammonia production, approximately 30% of emissions originate from reformer flue gas, while the remaining 70% of CO_2_ is inherently separated during syngas processing to purify the hydrogen. As a result, only compression is required for the utilization or storage of this CO_2_ stream. As described in our previous study,^10^ direct process emissions can be mitigated with near-commercial alternatives by capturing CO_2_ from flue gas, transporting CO_2_ from both syngas and flue gas for long-term storage, or replacing fuel-fired furnaces with electric alternatives. In this work, we consider the installation of a flue gas carbon capture (CC) plant as an extension to the existing facility without requiring modifications to other process sections. For the CO_2_ separated from syngas in the (electric) SMR, we account for the additional energy required for compression, ensuring its suitability for transport and storage. Note that in our model, the CO_2_ present in the syngas is always separated and compressed by default. Electrification of naphtha cracking is modeled by replacing the conventional furnace with an electric one while keeping the downstream olefin separation section unchanged. Similarly, electrifying ammonia production requires process modifications in the syngas section of the plant, while the HB section remains unchanged. Additionally, we introduce the option to divert syngas from the electric SMR before the WGS for use in methanol-based pathways—a feature not considered in our prior studies. Finally, we include water electrolysis to provide the hydrogen for ammonia synthesis. Further technical details on conventional processes, CO_2_ capture from flue gas, and electrification of high-temperature heat are discussed in Tiggeloven et al.^10^

### Alternative routes

In this study, we include several alternatives to fossil feedstock: plastic waste, multiple bio-based feedstocks, and CO_2_ from direct air capture (DAC) or biomass. Specifically, we consider (1) methanol-based olefin production via (1a) reverse water gas shift (rWGS) and (1b) plastic waste gasification, and (1c) direct CO_2_ hydrogenation to methanol, (2) hydrocarbon upgrading through (2a) ethanol dehydration (EDH) and (2b) propane dehydrogenation (PDH), and (3) electrochemical ethylene production via direct CO_2_ reduction. In the following, we provide a brief description of each alternative pathway; all cost and performance data are provided in Tables S1 and S2 in the supplemental information (SI).

#### Methanol-to-olefins

The methanol-to-olefins (MTO) process is a catalytic reaction that converts high-purity methanol into light olefins at temperatures of 350°C–500°C and pressures of 1–5 bar. It is a well-established commercial technology, with multiple industrial-scale plants in operation, primarily due to its ability to produce chemicals from coal.^17^ The process is highly exothermic, enabling efficient energy integration through waste heat recovery, which can generate low-pressure steam for use in product separation. The cost and performance data used in this study are derived from Ding et al.,^18^ which aligns closely with findings from other sources, such as Allgoewer et al.^19^

#### Methanol production

This study examines methanol production via three routes: (a) syngas production from CO_2_ via the rWGS reaction, (b) syngas production from mixed plastic waste (MPW) gasification, and (c) direct methanol synthesis from CO_2_ and H_2_ (only for the mid and long-term scenarios). The CO_2_ utilized in the rWGS and direct synthesis routes can be captured locally or imported, with imported CO_2_ sourced from DAC. The MPW composition is based on the composition of a low-quality mixed-plastic sorting residue.^20^

The cost and performance data for the rWGS route are based on the work of Ding et al.,^18^ using a state-of-the-art process configuration. Direct CO_2_ hydrogenation to methanol is technically feasible; however, commercial methanol synthesis catalysts, which are optimized for CO-rich feed gas, exhibit lower performance with CO_2_-rich feed gas.^21^ Given the ongoing research to enhance catalyst efficiency and overall process performance,^22^ we include this route in the mid- and long-term scenarios. In this study, we assume a Cu-ZnO-Al_2_O_3_ catalyst operating at approximately 250°C and 80 bar, following the design outlined in Allgoewer et al.^19^ Compared with the rWGS route, the lower reactor temperature allows heating with high-pressure steam instead of fuel combustion, potentially improving process integration and efficiency.

MPW gasification employs an indirectly heated gasifier, where methane and other recycled gases from the distillation section are combusted to provide the necessary heat for syngas production. A key advantage of this solution is its feedstock flexibility, minimizing the need for sorting and washing plastics, as the exact polymer composition does not dictate the process design. Although currently at the pilot scale, MPW gasification is considered a near-commercial technology. Cost and performance data are based on Afzal et al.,^23^ which describes a process involving feedstock shredding, gasification, syngas conditioning, and methanol synthesis at approximately 220°C and 80 bar. The process also generates low-pressure steam, which is reused internally. Similar to naphtha cracking and SMR, we incorporate the option to implement CO_2_ capture from the flue gas of MPW (heat provision side), utilizing cost and performance data from Weimann et al.^24^

#### Hydrocarbon upgrading processes

In this work, we consider standalone bio-EDH and bio-PDH, which are commercially viable processes that target specific ethylene and propylene, respectively. Cost and performance data are based on the process design by Ding et al.,^18^ while for the PDH, we consider the membrane-enhanced reactor design described as Case B in Brencio.^25^

#### Electrochemical processes

This study considers electrochemical processes for the direct production of (a) hydrogen and (b) ethylene. For the former, we consider an alkaline electrolysis cell (AEC) system, which can replace conventional syngas production in ammonia synthesis or provide hydrogen for methanol production. The technology is commercially available and is expected to scale rapidly in the coming decade.^26^ Details on electrolyzer type selection, as well as cost and performance data, are provided in previous work.^10^ Additionally, we explore the electrochemical reduction of CO_2_ to ethylene, an emerging technology at low TRL. While selectivity, catalyst stability, and carbonate formation, particularly in single-step electrolyzers, remain challenging, recent advancements highlight the potential of tandem CO_2_ reduction electrolyzers, which first convert CO_2_ to CO and subsequently CO to ethylene in a solid oxide electrolyzer (SOEC). This design mitigates CO_2_ losses to carbonate and leverages the high energy efficiency and geometric current densities of SOECs, offering a promising pathway for ethylene production. Here, we adopt optimistic cost and performance estimates for this tandem system based on Sisler et al.^27^

#### Dynamic operation and storage

With the exception of the CC, MPW-to-methanol and hydrocarbon upgrading routes, most alternative chemical production pathways require substantial amounts of low-carbon electricity or green hydrogen, which itself depends on low-carbon electricity. The variability of renewable-based electricity necessitates either energy storage or the dynamic operation of chemical processes with material storage. Our previous work examined the dynamic operation of electric cracking plants^28^ and electric SMR.^10^ However, data on the dynamic operation of alternative routes, especially of low TRL technologies, is scarce. To address this, we adopt the dynamic behavior of the Air Separation Units (ASUs) and HB processes, reported in the work of Armijo and Philibert.^29^ Moreover, we include several options for storage of electricity, intermediates, and products (ethylene, propylene, ammonia, and hydrogen). The Tables S3 and S4 summarize the dynamic performance parameters of all processes and the storage technology costs and performance data as detailed in our prior work.^10^

### Cluster case study

**Figure 2:**
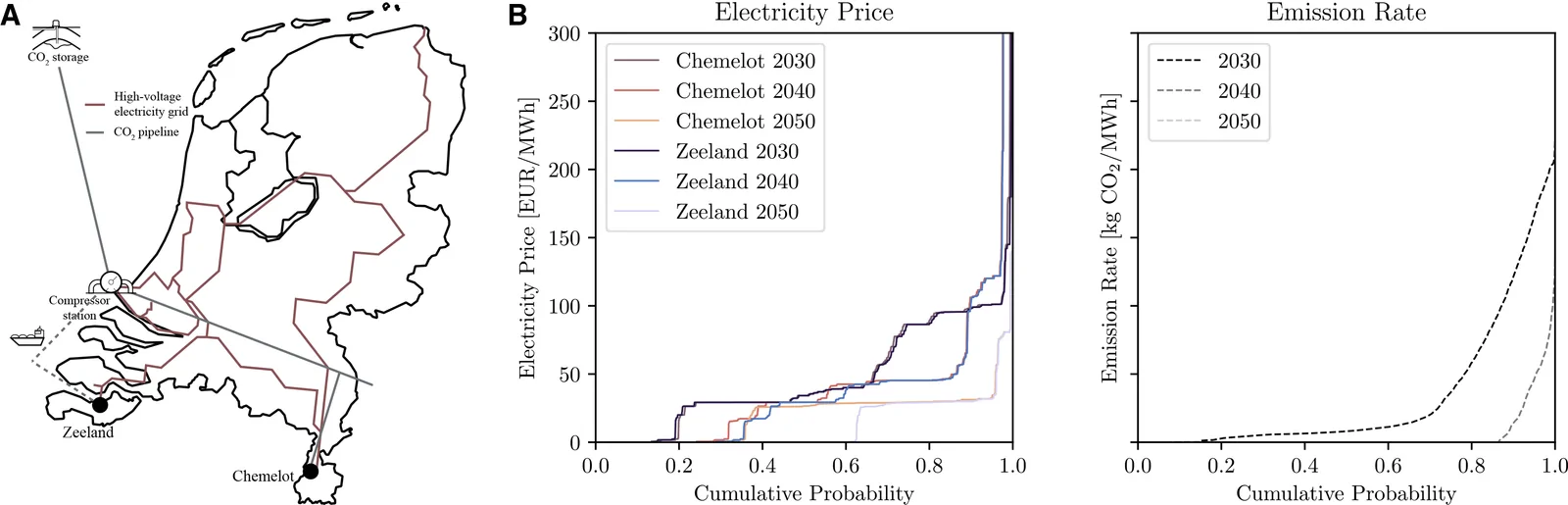
Overview of the industrial clusters and the associated electricity prices and grid emission rates (A) Map of the Netherlands showing the Chemelot and Zeeland clusters, CO_2_ transport routes, compression station, and storage site. (B) Cumulative distributions of electricity prices (middle) and grid emission intensities (right) for Chemelot and Zeeland under the 2030, 2040, and 2050 scenarios. Note that the distributions include loss-of-load hour costs exceeding 9,000 €/MWh, which occur in 0.3%–0.6%, 2.3%–2.5%, and 0% of the hours in 2030, 2040, and 2050, respectively.

We analyze the transition of two existing Dutch chemical clusters, Chemelot and Zeeland, toward net-zero chemical production. The Chemelot cluster is used as a baseline case study, while with Zeeland we test the effects of different locations, production volumes, access to relevant networks. Figure 2A shows the cluster locations within the Netherlands. Both clusters produce fertilizers and olefins, with ammonia, ethylene, and propylene as the primary platform chemicals, forming the basis for various downstream products. The two clusters were selected for several reasons: (1) they represent modern, integrated chemical clusters that produce bulk chemicals in large volumes; (2) specific process data are available from the Manufacturing Industry Decarbonisation Data Exchange Network (MIDDEN) initiative; (3) the key production lines are operated by different industrial actors but are integrated from an energy system perspective, representing a good example for coordinated transition; and (4) they are large emitters relevant for national and European emissions reduction pathways.

Given the high energy and carbon intensity in the production of these three platform chemicals, this analysis focuses exclusively on the transition of olefin and fertilizer production, excluding by-products and downstream processes. For olefins, we focus on ethylene and propylene, allowing a flexible propylene-to-ethylene (ratio) (P/E) demand ratio to accommodate shifts in production via alternative processes. By-products from the current conventional process, such as butene, butadiene, pentane, and aromatics, are excluded from the analysis under the assumption that their demand will adjust based on availability. In other words, we take the perspective that changes in the main production processes will drive downstream adaptations. For fertilizers, we treat downstream processes as fixed in terms of CO_2_ (for urea production) and energy demand. The energy and material consumption of all downstream processes are incorporated as fixed hourly demands,^30^^,^^31^^,^^32^ assuming that the total energy demand remains largely stable despite potential shifts in products. Table 1 presents the nameplate capacities of conventional ammonia and ethylene production plants, along with the energy and CO_2_ consumption of their downstream processes. Cluster data are obtained from the MIDDEN database and from our previous work.^10^^,^^30^^,^^31^^,^^32^^,^^33^

**Table 1:** Regional input data for Chemelot and Zeeland

**Node**	Nameplate capacities						
	Ammonia production [kt/y]	Ethylene production [kt/y]	Propylene production [kt/y]	CO_2_ utilization [kt/y]	Downstream steam consumption [TJ/y]	Downstream electricity consumption [TJ/y]	Downstream fuel gas consumption [TJ/y]
Chemelot	1,184	1,314	591	383	–	2,580	218
Zeeland	1,819	1,822	820	901	9,642	1,890	–

	Node-specific data			
	Industry area [km^2^]	Distance to harbor$\left(d_{h}^{\text{harbor}}\right)$ [km]	CO_2_ T&S costs $\nu_{{CO}_{2}}$ [€/tCO_2_]	CO_2_ price(*θ*) [€/tCO_2_]
Chemelot	8.00	230	111.51	150.31
Zeeland	5.07	100	98.42	150.31

	CO_2_ transport capacity [t/h]			Electricity grid capacity [MW]		
	Short-term	Mid-term	Long-term	Short-term	Mid-term	Long-term
Chemelot	114	114	148	750	2,000	2,500
Zeeland	285	354	742	1,900	3,250	4,600

The data include (1) nameplate capacities of conventional ammonia and olefin plants, and downstream energy and CO_2_ demand (excluding primary process energy); (2) CO_2_ T&S costs, onshore transport distances, and price assumptions; and (3) maximum CO_2_ export and electricity grid capacities across short-, medium-, and long-term scenarios. Downstream demand data from Batool and Wetzels,^30^ Oliveira and Dril,^31^ and Eerens and Dam^32^.

#### Temporal electricity data

Our modeling framework focuses on the local cluster level; therefore, hourly electricity prices and emission rates for the imported electricity are exogenous. Obtaining accurate forecasts for these data is complex, as they are time and space-specific and depend on the design of the future energy system, expected demand, and electricity grid, which remains uncertain. To provide reliable inputs, we adopt projected data from an hourly resolved electricity system model developed by the Dutch Organization for Applied Scientific Research (TNO).^34^ We use 2030 projections for the short-term, 2040 projections for the mid-term, and 2050 projections for the long-term scenario. The model incorporates nodal pricing to capture potential grid congestion and utilizes predictions aligned with the IP2024 Klimaat Ambities for 2030,^35^ the II3050 scenarios for 2040 and 2050,^36^ and the TYNDP 2024 National Trends and Global Ambitions scenarios for 2030/2040 and 2050,^37^ respectively. Figure 2B displays the electricity price distributions for the Chemelot and Zeeland regions, along with the associated grid emissions profile. Figure S1 in the SI shows the full hourly price and emission rate profiles for both clusters.

#### Feedstocks and products

The cluster imports feedstocks and energy carriers that can not be produced internally, supplies its main products to the downstream demand, and exports by-products that are not utilized within the cluster. Table 2 lists all feedstocks and energy carriers that can be imported, with their respective constant market prices in the reference and optimistic bio-feedstock price scenarios. The cost of DAC-CO_2_ in the reference scenario is based on net removal cost projections from Sievert et al.,^38^ corresponding to cumulative deployment levels of 1, 500, and 1,000 Mt-CO_2_ per year in the short-, mid-, and long-term, respectively.

**Table 2:** The market prices of the fossil and bio-based feedstocks for both clusters, with optimistic price scenarios in brackets

	Unit	Short-term	Mid-term	Long-term
DAC-CO_2_	€2022/t	618 (355)	475 (275)	355 (200)
Methane	€2022/MWh	56 (56)	56 (56)	59 (59)
Bio-Methane	€2022/MWh	141 (113)	141 (85)	148 (59)
Naphtha	€2022/t	732 (732)	732 (732)	732 (732)
Bio-Naphtha	€2022/t	1,830 (1,464)	1,830 (1,098)	1,830 (732)
Bio-Ethanol	€2022/t	1,734 (1,387)	1,734 (1,040)	1,734 (694)
Bio-Propane	€2022/t	1,473 (1,178)	1,473 (884)	1,473 (589)
MPW	€2022/t	780 (780)	780 (780)	780 (780)

All feedstocks are assumed to be available without supply constraints, except for MPW, which is limited to reflect plastic recycling limitations. More specifically, the maximum plastic recycling rate is set at 75% in the short term, increasing linearly to 94% in the long term, in line with estimates from Bachmann et al.^39^ This constraint accounts for the inherent carbon losses in plastic recycling, which reduce the available stock with each cycle. While bio-based feedstocks are indeed subject to global supply limitations, we assume that availability at the cluster level is unconstrained, with potential scarcity reflected through higher prices rather than import restrictions.

Future chemical demand is highly uncertain, influenced by factors such as population growth, recycling rates, and (bio-)feedstock and product prices.^8^ As these macroeconomic drivers are beyond the scope of this study, we assume a constant demand for these chemicals across the short, mid, and long term, allowing us to focus on technology pathways and emission reduction strategies. This is also more consistent with a local brownfield cluster analysis because existing clusters are unlikely to significantly change their production capacity (unless market or regulatory conditions become unfavorable). Consequently, the hourly demand is based on the nameplate capacities of the clusters, as presented in Table 1.

#### Critical infrastructure

Although both clusters currently utilize similar production processes for the same platform chemicals, notable differences in critical infrastructure availability exist, which may influence the optimal pathways for transitioning to net-zero chemical production. Zeeland, situated along the North Sea coast, benefits from better access to CO_2_ pipelines and grid connections, while Chemelot, being landlocked, faces more restricted infrastructure access. Table 1 presents anticipated grid connections and CO_2_ pipeline capacities for the short, medium, and long term, as outlined in the Integral Infrastructure 2030–2050 study^36^ and the cluster’s Cluster Energy Strategy documents.^40^^,^^41^^,^^42^^,^^43^ For each cluster, we calculate the unitary CO_2_ transport and storage (T&S) costs as:

(Equation 1) $$\nu_{{\text{CO}}_{2},d}={\text{c}}^{\text{T\&S}\;\text{offshore}}+{\text{c}}^{\text{transport}\;\text{to}\;\text{harbor}}{\text{d}}^{\text{harbor}}$$

where $\nu_{{\text{CO}}_{2},d}$ denotes the cost per ton of CO_2_ transported to offshore storage. The offshore T&S tariff, c^T&S offshore^, is set at 90.60 €/tCO_2_ based on Aramis project data from the Dutch Ministry of Economic Affairs and Climate Policy report,^44^ assuming gas transport from the harbor to offshore storage. For CO_2_ transport from the clusters to the harbor, we assume transport by onshore pipeline for Chemelot and by ship at 7 barg for Zeeland. The specific transport costs, ${\text{c}}_{h}^{\text{transport}\;\text{to}\;\text{harbor}}$, are calculated using the unitary CO_2_ transport cost function from Oeuvray et al.^45^ The transport distances, $d_{h}^{\text{harbor}}$, and the resulting costs for both clusters are detailed in Table 1.

#### CO_2_ prices and emission limits

The Dutch Emissions Authority (NEA) has set an annual increase of €12.69 for the CO_2_ emission price, reaching €150.31 by 2030.^46^ While future emission prices are influenced by various uncertain factors, including policy changes, market demand, and transition pathways, it is reasonable to expect that the CO_2_ price will not fall below this rate. Accordingly, we assume a minimum CO_2_ price of €150.31 across all modeled decades. This carbon price drives emission reductions in the short term (2030), while for the mid- and long-term intervals, hard emission caps are enforced instead, as future CO_2_ price trajectories are highly uncertain. The mathematical formulation of both modeling strategies and the specific emission targets are described in the emission balance section.

### Methodological framework

In this study, we use an in-house, open-access MILP modeling framework (AdOpT-NET0),^16^ which has been used in several previous publications, including analysis at the cluster level.^10^ Here, we extend the framework in three key directions: (1) Expanding the time horizon to assess the long-term transition of a chemical cluster to net-zero emissions, (2) including brownfield pathways to explore the trade-offs between retrofitting existing clusters and developing new ones, and (3) incorporating additional technology and alternative feedstock pathways to tackle scope 3 emissions alongside scopes 1 and 2. It is worth noting that AdOpT-NET0 is a technology-focused energy system model, which allows to evaluate several technologies using fairly detailed models combined with time and space discretization; on the other hand, this set some limits on the broader context, for example, no market dynamics are included.

In the following sections, we provide the problem formulation, the implementation of brownfield optimization, and the calculation and integration of scope 1, 2, and 3 emissions within the model framework.

### Problem formulation

Here, we focus the discussion on the critical constraints added to simulate the cluster transition and the brownfield approach. For the interested reader, the full formulation of the model applied to chemical clusters and energy systems is reported in existing peer-reviewed publications.^10^^,^^16^^,^^28^^,^^47^ The general formulation of the MILP is as follows:

(Equation 2) $$\begin{array}{cc} \min_{\mathrm{v,w}} & \;f\left(v,w\right)\\ \;\text{s.t.} & \;Av+Bw=b\\ & \;v\geq 0\in R^{N_{v}},w\in {\left\{0,1\right\}}^{N_{w}} \end{array}$$

where *f* represents the objective function, with *v* and *w* denoting continuous and binary decision variables, respectively. **A** and **B** define the coefficients of the system, **b** is the right-hand-side parameter vector, and *N* specifies the number of continuous and binary variables. Binary variables *w* capture discrete decisions such as the on/off operation of a technology or the selection and installation of a new unit, while continuous variables *v* represent quantities such as technology sizes, energy and material demands, production and consumption rates, and import/export flows. The equality constraints **A***v* + **B***w* = **b** encode the physical and operational logic of the system, including mass and energy balances, technology performance characteristics, and operational constraints. The proposed framework simultaneously optimizes design and operational decisions. Design variables define process and technology selection as well as capacity sizing, while operational variables captur [0-9] e key aspects varying in time, such as energy and material flows and storage levels. The operational time horizon covers 8,760 h in each optimization, aggregated into 10 representative design days (further details on the selection of design days are provided in the SI). At full temporal resolution, the model comprises approximately 5.5–7.5 million continuous variables and 280,000 integer variables. After temporal aggregation, following the approach described in Gabrielli et al.,^48^ the model size varies depending on the scenario and year, ranging from 5.5 to 7.4 million continuous variables and from 6,700 to 11,500 integer variables. The optimization problem is solved using Gurobi v12.0 with a maximum MIP gap of 1.0%.^49^ The total computing time on a machine equipped with a QEMU Virtual CPU version 2.5+ was approximately 48 h, with the short-term analysis taking 5 h, the mid-term 30 h, and the long-term 13 h. The computations utilized up to 12 threads on a system with 48 physical cores and 48 logical processors.

#### Objective function

The objective function is the total annuitized system cost for each decade interval d∈D:

(Equation 3) $$C_{d}=C_{d}^{\text{CAPEX}}-C_{d}^{\text{decommission}\;\text{value}}+C_{d}^{\text{import}}+C_{d}^{{\text{CO}}_{2}storage}+C_{d}^{{\text{CO}}_{2}emission}$$

We consider three independent optimization periods, 2030, 2040, and 2050, corresponding to the elements of D. In the brownfield case, information is transferred between these periods, as discussed in the following section. The total cost comprises capital expenditures $\left(C_{d}^{\text{CAPEX}}\right)$, which is offset by the residual value of decommissioned assets $\left(C_{d}^{\text{decommission}\;\text{value}}\right)$, and operating expenditures. The latter include import costs for fuels and feedstocks $\left(C_{d}^{\text{import}}\right)$, costs for CO_2_ transport and storage $\left(C_{d}^{{\text{CO}}_{2}storage}\right)$, and expenses due to CO_2_ emissions pricing $\left(C_{d}^{{\text{CO}}_{2}emission}\right)$.

The annualized capital costs are as follows:

(Equation 4) $$C_{d}^{\text{CAPEX}}=\underset{k\in K}{\sum }u_{k,d}\left(\psi_{k,d}+\omega_{k,d}\right)\left(\lambda_{k,d}S_{k,d}+\zeta_{k,d}\right)$$

Here, k∈K⊂M includes newly installed technologies, *S*_*k*,*d*_ is the size of technology *k* in decade *d*, *λ*_*k*,*d*_ and *ζ*_*k*,*d*_ are size-dependent and size-independent cost parameters, respectively, *ω*_*k*,*d*_ is the annuity factor, and *u*_*k*,*d*_ ∈ {0, 1} is a binary variable indicating whether technology *k* is installed in decade *d*. The size-independent cost *ζ*_*k*,*d*_, multiplied by the binary variable *u*_*k*,*d*_, introduces a fixed installation cost that is incurred only when a technology is selected, while *λ*_*k*,*d*_*S*_*k*,*d*_ captures the additional size-dependent cost. Together, these two terms approximate economies of scale: installing a plant entails a large upfront fixed cost alongside a variable capacity cost, reflecting the fact that industrial-scale plants are typically deployed at large capacities rather than incrementally expanded. This formulation avoids the need for piecewise approximations, which are more complex to implement, while still capturing the key cost structure of capacity expansion decisions. Note that the product *u*_*k*,*d*_*S*_*k*,*d*_ is bilinear; linearity is restored by applying a big-M reformulation, which linearizes the product of the binary and continuous variables through a set of linear inequality constraints.

Maintenance costs, *ψ*_*k*,*d*_, are a fraction of the total capital costs. Cost parameters for all processes and storage technologies are provided in Tables S1 and S2. The decommissioning value of assets is as follows:

(Equation 5) $$C_{d}^{\text{decommission}\;\text{value}}=\underset{l^{\prime }\in L^{\prime }}{\sum }\xi_{l^{\prime },d}\left(S_{l^{\prime },d-1}-S_{l^{\prime },d}\right)\omega_{l^{\prime },d}$$

Here, $L^{\prime }\;\subset \;L\;\subset \;M$ is the set of conventional existing plants, *S*_*l*′,*d*−1_ is the legacy plant size, *S*_*l*′,*d*_ is the optimized size, and *ξ*_*l*′,*d*_ denotes the value of the existing assets.

Import costs are calculated for fuels and feedstocks that cannot be produced within system boundaries and which are purchased at market prices, such as electricity, methane, naphtha, MPW, and bio-based feedstocks:

(Equation 6) $$C_{d}^{\text{import}}=\underset{n\in N}{\sum }\overset{T}{\underset{t=1}{\sum }}\left(\iota_{n,t,d}I_{n,t,d}\right)$$

where N denotes the carriers, *I*_*n*,*t*,*d*_ is the imported quantity, and *ι*_*n*,*t*,*d*_ is the hourly price. Fuel and feedstock prices remain constant within each decade but can vary between decades, while electricity prices vary by timestep and decade.

The CO_2_ transport and storage costs are as follows:

(Equation 7) $$C_{d}^{{\text{CO}}_{2}storage}=\overset{T}{\underset{t=1}{\sum }}\left(\nu_{{\text{CO}}_{2},d}J_{{\text{CO}}_{2},t,d}\right)$$

where $J_{{\text{CO}}_{2},t,d}$ is the exported and stored CO_2_ amount, and $\nu_{{\text{CO}}_{2},d}$ is the transport and storage cost per ton.

#### Energy and material balance constraint

To ensure the energy and material balance of the cluster is maintained for each carrier *n* and hour *t*, the balance equation is expressed as:

(Equation 8) $$\underset{m\in M}{\sum }\left(P_{m,n,t,d}-F_{m,n,t,d}\right)+I_{n,t,d}-J_{n,t,d}-D_{n,t,d}=0$$

where M is the set of all technologies (existing and new). *P*_*m*,*n*,*t*,*d*_ and *F*_*m*,*n*,*t*,*d*_ represent the output and input of technology *m*, respectively. *I*_*n*,*t*,*d*_ and *J*_*n*,*t*,*d*_ are the cluster’s imports and exports across system boundaries, and *D*_*n*,*t*,*d*_ denotes the total demand of all plants.

#### Performance constraints of industrial processes

Following previous work,^10^^,^^28^ each industrial process is modeled as an input/output conversion technology. While all technologies share the same type of conversion performance constraints, their conversion efficiencies are represented by individual linear approximations. Dynamic operation is restricted by factors such as operation envelope (OE) and ramping time (RT), but the conversion efficiency remains constant across part-load and full-load operations. The (dynamic) performance parameters for all conversion technologies and processes are provided in the SI in Tables S1 and S4.

The production (*P*) and consumption (*F*) of chemical processes are determined as:

(Equation 9) $$P_{m,n^{\prime },t,d}=\alpha_{m,n^{\prime },d}F_{m,n_{\mathrm{main}},t,d}$$

(Equation 10) $$F_{m,n^{\prime \prime },t,d}=\beta_{m,n^{\prime \prime },d}F_{m,n_{\mathrm{main}},t,d}$$

where *α*_*m*,*n*′,*d*_ and $\beta_{m,n^{\prime \prime },d}$ represent the conversion efficiency for outputs $n^{\prime }\in N^{\prime }\subset N$ and the input ratio for $n^{\prime \prime }\in N^{\prime \prime }\subset N$, respectively. Although the framework supports interval-varying *α*_*m*,*n*′,*d*_ and $\beta_{m,n^{\prime \prime },d}$ (e.g., for technological learning), they are assumed constant in this study to keep the computational complexity reasonable.

The installed capacity of chemical processes and storage technologies is constrained as follows:

(Equation 11) $$S_{m}^{\text{min}}\leq S_{m,d}\leq S_{m}^{\text{max}}$$

where *S*_*m*,*d*_ is the installed capacity of technology *m* in decade *d*, and $S_{m}^{\text{min}}$ and $S_{m}^{\text{max}}$ are the respective minimum and maximum capacities.

Technology operation is limited to the OE or complete shutdown, expressed by:

(Equation 12) $$\gamma_{m,n_{\mathrm{main}}}\;x_{m,t,d}\;S_{m,d}\leq F_{m,n_{\mathrm{main}},t,d}\leq x_{m,t,d}\;S_{m,d}$$

where *x*_*m*,*t*,*d*_ ∈ 0, 1 indicates the on/off status, and $\gamma_{m,n_{\mathrm{main}}}$ is the minimum operating fraction of the installed capacity *S*_*m*,*d*_.

The RT constraints, representing the time to transition between idle and full capacity, are given by:

(Equation 13) $$-\frac{S_{m,d}}{RT}\leq F_{m,n_{\mathrm{main}},t,d}-F_{m,n_{\mathrm{main}},t-1,d}\leq \frac{S_{m,d}}{RT}$$

We categorize conversion technologies into three types based on their dynamics: (1) slow technologies, (2) medium-dynamic technologies, and (3) fast technologies. Slow technologies, often conventional, have a small OE, limited ramping capabilities, and are unable to start up or shut down production. Medium-dynamic technologies typically achieve an OE above 50% and can start up and shut down production. Ramping constraints may apply depending on the specific technology. Fast dynamic technologies have no constraints on OE or ramping behavior. Additionally, storage technologies involve unique performance considerations. The detailed operational constraints for each technology category, including dynamic operation and storage constraints, are provided in the SI.

We categorize conversion technologies into three types based on their dynamics: (1) slow technologies, (2) medium-dynamic technologies, and (3) fast technologies. Slow technologies, often conventional, have a small OE, limited ramping capabilities, and are unable to start up or shut down production. Medium-dynamic technologies typically achieve an OE above 50% and can start up and shut down production. Ramping constraints may apply depending on the specific technology. Fast dynamic technologies have no constraints on OE or ramping behavior. Additionally, storage technologies involve unique performance considerations. The detailed operational constraints for each technology category, including dynamic operation and storage constraints, are provided in the SI.

#### Brownfield analysis

**Figure 3:**
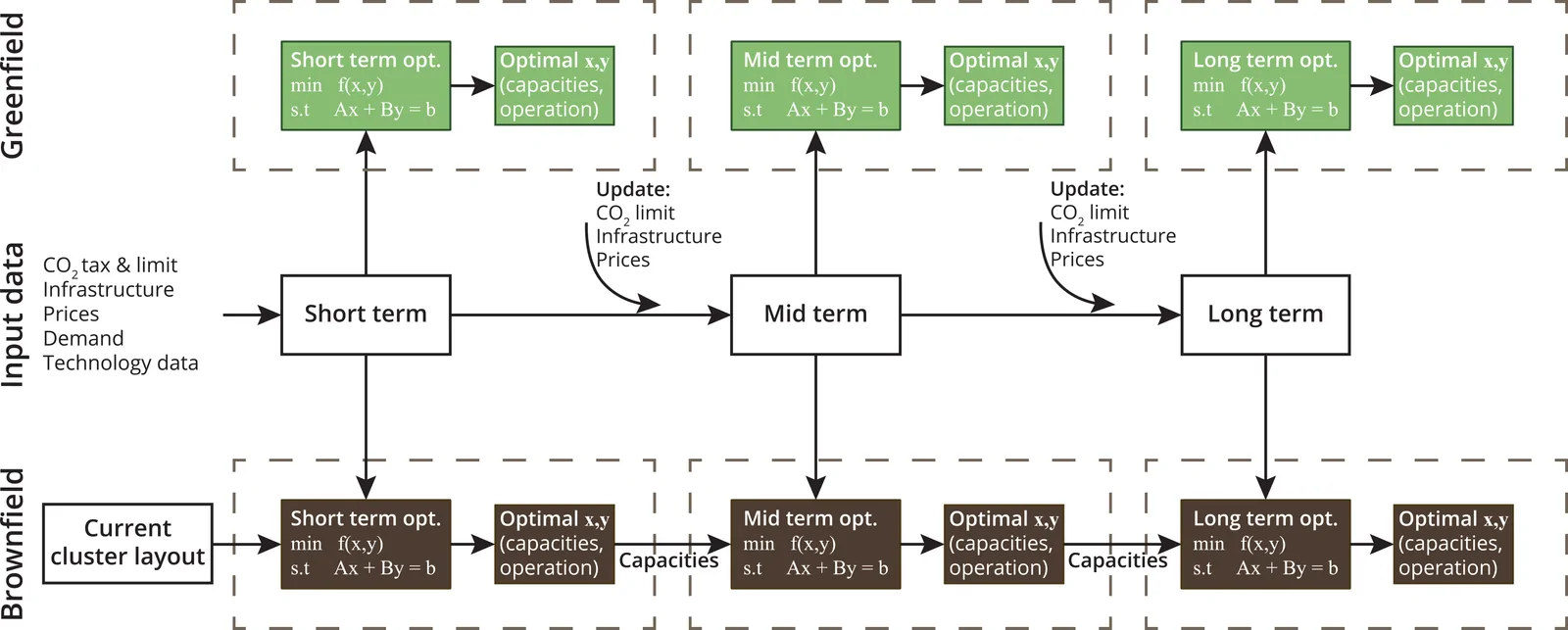
Simplified algorithm diagram illustrating the computational flow for the greenfield approach (top, in green) and the brownfield approach (bottom, in brown)

The transition of the chemical industry to net-zero must be timely, yet it is expected to unfold over multiple decades; as a result, it is subject to significant uncertainty, such as future technology developments, policy changes, and market conditions. In practice, investment decisions, especially when involving large expenditures, are made dynamically over time as new information emerges.^14^ Various approaches exist to represent decision-making and information availability over long time horizons in optimization models, including (1) perfect foresight, where all future information is assumed to be known for the entire optimization period; (2) myopic foresight, in which decisions are optimized for each investment period independently, without anticipating future changes, and results are carried forward to subsequent periods; and (3) a rolling horizon approach, where the optimization is performed sequentially over overlapping time intervals, updating decisions as new information becomes available at each step.^50^ A key decision for chemical companies is whether to build net-zero clusters as greenfield projects or to gradually retrofit existing plants over time. In most OECD countries, as well as for those with a well-established chemical industry, the latter brownfield approach is likely to dominate. Therefore, including brownfield options is particularly important for studies that aim at identifying optimal transition pathways. As shown in Figure 3, in this work, we adopt a brownfield approach where the model optimizes the transition of an existing chemical cluster toward a long-term net-zero design by sequentially optimizing three decade-long intervals: the short term (starting in 2030), mid term (2040), and long term (2050), each with increasingly stringent emission constraints. During the full time horizon, the model sees updated boundary conditions at the beginning of each decade, such as the availability of CO_2_ infrastructure, grid capacity, and the supply and cost of green electricity. This brownfield approach is compared with the results of a more traditional greenfield approach.

Starting from the cluster’s current configuration, investment and operational decisions are allowed at the beginning of each decade. Because of the MILP problem size and complexity, the model optimizes operational decisions for the first year of each interval, assuming that the resulting operational strategy remains unchanged for the remainder of the decade. The model assumes perfect foresight within each interval (e.g., for hourly price variations), but is myopic with respect to the next period, i.e., decisions are based solely on the conditions of the current decade without considering future developments.

For each decade, the capacities of newly installed technologies k∈K are optimized and carried forward as existing plants l∈L in the subsequent decade period. The inter-temporal coupling between decade intervals is enforced through the following carry-over constraint, which ensures that capacities installed in one decade are inherited as existing assets in the next:

(Equation 14) $$S_{k,d-1}=S_{l,d}$$

This myopic structure can result in path-dependency and lock-in effects, as decisions made in earlier intervals constrain the feasible solution space of subsequent ones.

The greenfield analysis likewise optimizes the system over three time periods. However, unlike the brownfield case, it assumes no pre-existing infrastructure, with all capacity investments made upfront at the start of the decade. Consequently, the greenfield scenario is unconstrained by legacy assets, allowing its results to serve as a benchmark for comparison with the brownfield analysis.

Two factors become particularly important: (1) the technical lifetime of a technology and (2) the depreciation period of plants. The former is critical when evaluating situations where existing facilities reach the end of their technical lifetime, requiring decisions about their maintenance or replacement. The latter, which represents the accounting time frame over which the asset loses value for tax purposes, is used to calculate annuitized investment costs. Often, in academic literature, the two are assumed to be equal; however, in reality, technical lifetimes frequently exceed depreciation periods, leading to scenarios where only operating costs remain after depreciation. Furthermore, technical lifetimes can often be extended through increased maintenance expenditures.

Within this framework, a key challenge arises when a plant becomes economically unsustainable before reaching the end of its depreciation period or technical lifetime. In such cases, a decision must be made whether to continue operations or to decommission the plant. In reality, operators may delay decommissioning due to the irreversible nature of the decision and the possibility of future economic recovery, such as cost reductions or revenue increases. In the optimization model, postponing decommissioning is not an option, as the cost-minimizing objective function inherently favors immediate replacement whenever more cost-effective alternatives are available. Moreover, since the model lacks foresight beyond the current interval, it cannot anticipate future economic conditions. As the remaining depreciation value must be paid regardless of whether the plant is decommissioned, investment costs of existing plants are considered sunk costs. As a result, they should not be included in the objective function, ensuring that decisions focus solely on whether and how to operate existing plants efficiently, without being influenced by past investments. To reflect this, we use an annuitized investment cost $C_{d}^{\text{CAPEX}}$ in the objective function (Equation 4) that accounts only for newly installed technologies k∈K, excluding existing plants. Instead, to account for the total economic burden of the system, we include all investments, both new and prior, in a post-processing step. Accordingly, the total annuitized cluster production costs for interval *d*, denoted as *TCPC*_*d*_, is calculated as:

(Equation 15) $$TCPC_{d}=\frac{\overset{d}{\underset{i=1}{\sum }}\sigma_{i}C_{i}^{\text{CAPEX}}-C_{d}^{\text{decommission}\;\text{value}}+C_{d}^{\text{import}}+C_{d}^{{\text{CO}}_{2}storage}+C_{d}^{{\text{CO}}_{2}emission}}{\underset{n\in N}{\sum }\overset{T}{\underset{t=1}{\sum }}D_{n,t,d}}$$

Here, $\sum_{i=1}^{d}\sigma_{i}C_{i}^{\text{CAPEX}}$ represents the annuitized investment costs that are still within their depreciation periods at the start of interval *d*, and includes both new investments and the remaining depreciation of existing assets. The correction factor *σ*_*i*_ accounts for the remaining depreciable value of prior investments at the start of interval *d*, and is defined as:

(Equation 16) $$\sigma_{d}=\frac{min\left({\text{LT}}_{d},\Omega_{d}\right)}{\Omega_{d}}$$

where LT_*d*_ denotes the remaining depreciation lifetime of investments at the start of interval *d*, and Ω_*d*_ is the duration of the interval.

Another factor that characterizes long-term investment decisions is the timing of investments relative to construction time: even after a final investment decision is taken, a few years might be required before plants become operational. This factor depends on several local and international conditions (e.g., supply chain constraints, permitting, social acceptability, technology readiness level), and is inherently difficult to embed in a deterministic optimization model. Moreover, it would require additional inter-temporal constraints that link investment decisions to future operational periods, further exacerbating the solution complexity. Accordingly, in this work, we neglect this behavior and consider new plants to be operational at the beginning of each optimization interval.

To represent the current clusters in the optimization, we include the existing ammonia and ethylene production facilities, see Table 1 and Batool and Wetzels,^30^ Oliveira and Dril,^31^ and Eerens and Dam^32^ as a subset of existing plants $l^{\prime }\in L^{\prime }$ in the short-term interval. Capacities are fixed to existing values. A 1% increase was required to prevent numerical issues in the model that could arise from rounding errors except for the HB process, which can be expanded to enable flexible operation. The existing plants $l^{\prime }\in L^{\prime }$ can be taken to the next time interval when decommissioning is not optimal. To account for operation beyond the typical technical lifetimes, which is common in large chemical facilities, we apply an arbitrary increase of operational expenditures (OPEX) (10%) and assume they can technically operate until the end of the analysis period (long-term).

When these conventional plants $l^{\prime }\in L^{\prime }$ are decommissioned, we consider that their balance-of-plant (BoP) components (here assumed as 30% of investment costs) can be repurposed for new facilities. This is also commonplace in large chemical factories. For simplicity, decommissioning benefits are annuitized using the same depreciation period and discount rate as the plant *l*′ itself. To ensure decommissioning entails shutting down the entire process rather than partial closure, we introduce the following constraint:

(Equation 17) $$S_{l^{\prime },d}=S_{l^{\prime },d}^{\text{initial}}g_{l^{\prime },d}$$

where *g*_*l*′,*d*_ ∈ 0, 1 is a binary decision variable that indicates whether the facility *l*′ is operational (*g*_*l*′,*d*_ = 1) or fully decommissioned (*g*_*l*′,*d*_ = 0).

For the remaining existing technologies l∈L, i.e., those newly installed during previous decade intervals, we assume they cannot be decommissioned. Consequently, these plants will always incur maintenance and associated OPEX, but they can suspend operations due to their generally higher flexibility. This simplification is justified by the fact that newly installed plants typically have technical lifetimes and depreciation periods that exceed the 30-year analysis horizon, meaning that none of these plants will reach the end of their lifetime within the modeled time frame. As a result, no inter-temporal constraints are needed to track lifetime expiry across decade intervals, which would otherwise significantly increase the computational complexity of the problem. Finally, in the brownfield analysis, we assume that new plants can be constructed without requiring the decommissioning or demolition of other plants, as spatial restrictions are highly specific to cluster and technology features.

#### Emission balance

**Figure 4:**
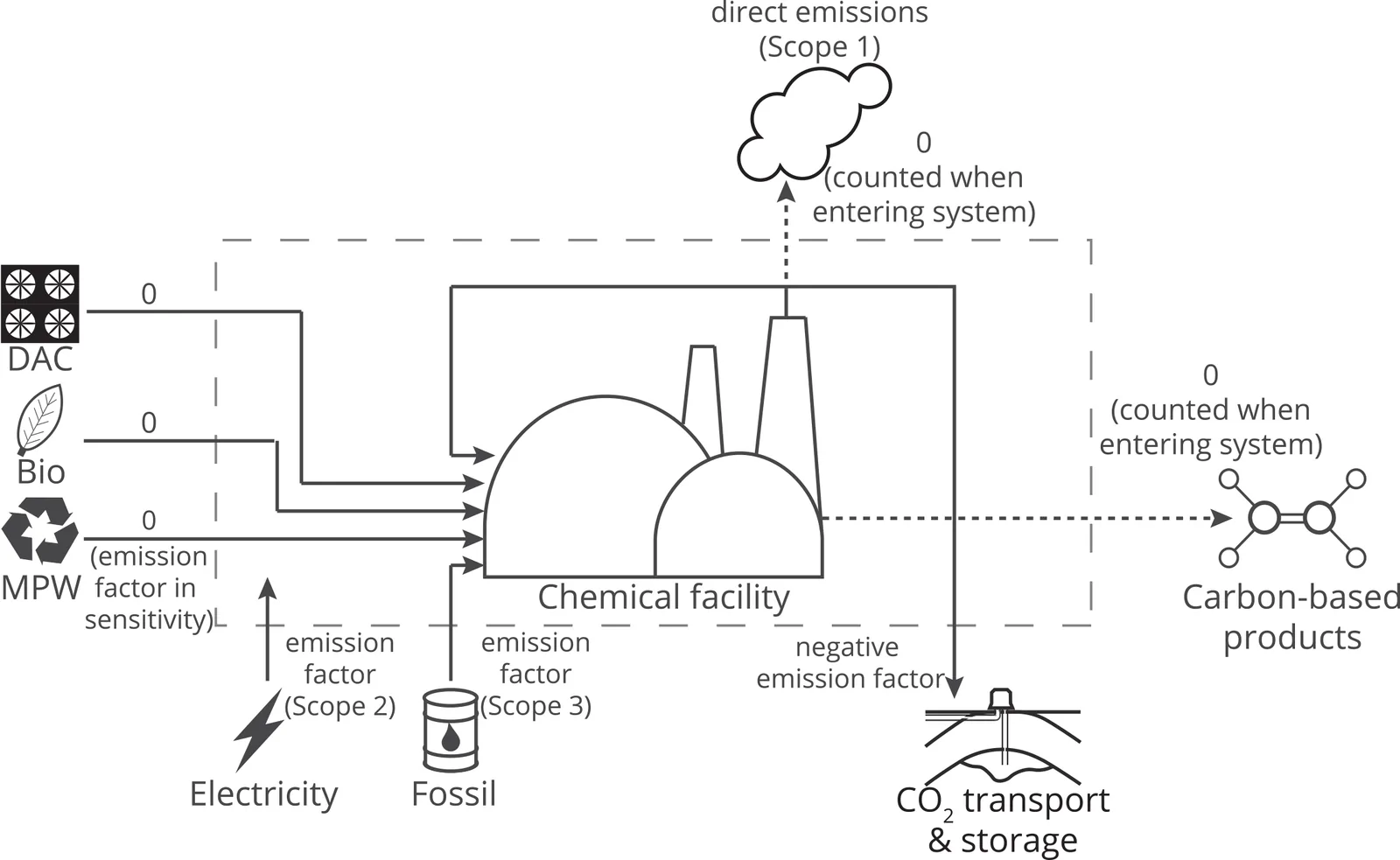
Emission balance of a generic chemical cluster, including scope 1, scope 2, and scope 3 emissions Negative emissions can occur coupling CO_2_ storage with capture from bio feedstock or from DAC.

The GHG emissions of a chemical cluster depend on both process characteristics and system boundaries, such as the carbon intensity of electricity and imported feedstocks. In this study, and as shown in Figure 4, we account for all emissions that can be controlled by the cluster actors, including (1) direct process emissions (scope 1), (2) indirect emissions from imported electricity (scope 2), and (3) emissions from product end-of-life (scope 3). It is important to note that, unlike higher-level models, cluster-level models do not explicitly capture land-use emissions and upstream and/or downstream emissions across the entire supply chain. While such emissions can be substantial,^51^ their mitigation largely depends on factors external to the cluster design and operation itself. Moreover, their offsetting is likely to happen outside the physical boundaries of the cluster. Therefore, they fall outside the scope of this study and are not considered in the emission balance. Consequently, scope 3 emissions in this work refer specifically to product end-of-life emissions, i.e., the emissions released upon combustion or decomposition of the chemical products manufactured within the cluster, and the term “net-zero” refers exclusively to the balance of scope 1, scope 2, and scope 3 emissions as defined earlier and within the defined cluster boundary. Nevertheless, the emission balance considered in this work allows for designing chemical clusters that would fit and align with a broad net-zero society. Consistently with this approach, DAC is not treated as a source of negative emissions but exclusively as a means to provide non-fossil carbon, thereby excluding questionable offsetting approaches, such as that of net-zero oil.^52^

Reducing end-of-life scope 3 emissions from a cluster perspective is challenging, as chemical producers have limited control over how their products are managed downstream. However, focusing solely on direct process emissions provides an incomplete picture, as many chemical products contain fossil carbon, which is likely released as CO_2_ at end-of-life. In the most optimistic scenario, carbon embedded in chemical products is fully recycled (e.g., plastic recycling) or permanently stored (e.g., combustion with CC and storage) without CO_2_ losses to the atmosphere. Conversely, in the most pessimistic–but more realistic–case, all embedded carbon is eventually released as CO_2_. While controlling end-of-life is hardly viable, chemical producers have full control over the type of carbon feedstock entering the systems: transitioning from fossil to bio-based or air-mined carbon feedstock means transitioning to net-zero (see Gabrielli et al.^48^ and Gabrielli et al.,^8^ for a detailed discussion on this topic). Regardless of downstream processes beyond the system boundaries, importing fossil-based feedstocks contributes to positive emissions, as well as importing electricity whenever the carbon intensity is positive.

In contrast, switching to bio-based or DAC-based carbon feedstocks does not result in positive emissions and is in line with a net-zero plant. On the other hand, assigning scope 3 emissions to MPW feedstock is less obvious, as it fundamentally depends on the origin of the recycled plastic, information that can be obtained only with national-level simulations. In this work, we consider as reference the case where the original and additional carbon input inherent to plastic recycling is provided by bio-based feedstocks or CO_2_ from DAC, meaning that all direct scope 1 and downstream emissions related to the use of MPW are zero. Since this assumption is optimistic and could significantly influence system design, we also test the case where MPW is assumed to have fossil origins. In this case, we apply a scope 3 emission factor of 2.38 tCO_2_/t of plastic, based on the carbon content of the waste after sorting but prior to agglomeration.^20^

Additionally, CO_2_ capture and storage can be adopted as a measure to decrease (or to go negative) CO_2_ emissions. Figure 4 provides an overview of the emission balance considered in this study. Overall, the total CO_2_ emissions are calculated as follows:

(Equation 18) $$E_{d}=\overset{T}{\underset{t=1}{\sum }}\left(\varepsilon_{\text{electricity},t,d}I_{\text{electricity},t,d}+\varepsilon_{n^{\prime \prime \prime },d}I_{n^{\prime \prime \prime },t,d}+\kappa_{{\text{CO}}_{2},d}J_{{\text{CO}}_{2},t,d}\right)$$

where $\varepsilon_{n^{\prime \prime \prime },t,d}$ is the standard CO_2_ emission factor in the Netherlands (obtained from Zijlema^53^) of subset of fossil-based feedstocks and fuels *n*′′′ ∈ *N*‴ ∈ *N*, *ϵ*_electricity,t_ is the hourly CO_2_ emission factor of the electricity grid, and $\kappa_{{\text{CO}}_{2},t,d}$ is the export emission factor of CO_2_ to long-term storage, which is −1. When allocating scope 3 emissions to MPW, the emission factor of the process is assigned to *ϵ*_*MPW,t,d*_. An overview of the emission factors for all feedstocks is provided in Table 3.

**Table 3:** Emission factors for imported fossil and bio-based feedstocks for both clusters

	Unit	Emission factor $\varepsilon_{n^{\prime \prime \prime },t,d}$
DAC-CO_2_	t/t	0
Methane	t/MWh	0.20
Bio-Methane	t/MWh	0
Naphtha	t/t	3.23
Bio-Naphtha	t/t	0
Bio-Ethanol	t/t	0
Bio-Propane	t/t	0
MPW	t/t	0 (2.38)

The MPW emission factor in brackets represents the scope 3 emissions of MPW, used in the sensitivity analysis.

To ensure that the model utilizes CO_2_ captured from DAC exclusively as a feedstock for carbon-based chemicals rather than for generating negative emissions (the adoption of DAC as purely a negative emission technology is out of scope), we impose the following constraint:

(Equation 19) $$\overset{T}{\underset{t=1}{\sum }}J_{{\text{CO}}_{2},t,d}\leq \overset{T}{\underset{t=1}{\sum }}P_{m^{\prime },{\text{CO}}_{2},t,d}$$

where $m^{\prime }\in M^{\prime }\subset M$ represents conversion technologies that generate CO_2_, explicitly excluding temporary CO_2_ storage. This ensures that only captured CO_2_ can be sequestered for negative emissions.

The expenses related to the CO_2_ emission price are calculated as

(Equation 20) $$C^{{\text{CO}}_{2}emissionprice}=\theta^{{\text{CO}}_{2}}E$$

where $\theta^{{\text{CO}}_{2}}$ represents the CO_2_ emission price for the system. Note that $\theta^{{\text{CO}}_{2}}$ carries no subscript *d*, reflecting the assumption that the same price floor applies across all decade intervals. It is important to note that the carbon price enters the objective function as a cost term, directly affecting production costs and driving emission reductions in the short-term without imposing a hard emission target. For the mid- and long-term intervals, relying solely on carbon pricing would introduce significant uncertainty into the objective function, as future CO_2_ prices are highly uncertain. Therefore, for the 2040 and 2050 scenarios, we instead enforce hard emission caps:

(Equation 21) $$E_{d}\leq E_{d}^{\max}$$

where $E_{d}^{\max}$ denotes the maximum allowable emissions in decade interval *d*. No limit is imposed in the short term, where emission reductions are instead driven by the CO_2_ price in the objective function. In the mid term, a 50% reduction relative to the brownfield short-term emissions is required, and in the long term, emissions must reach net zero. As a sensitivity analysis, we include one additional brownfield scenario with an emissions limit of 25% reduction in the short-term relative to the cost-optimal short-term emissions.

## Results

Here, we present the results of the optimization model; we first analyze the transition strategies for the chemical cluster of Chemelot, comparing greenfield and brownfield configurations under scope 1–3 constraints. Moreover, we examine the impact of (1) excluding scope 3 emissions, (2) enforcing stricter emission limits in 2030, (3) having improved infrastructure accessibility, (4) technology readiness of CO_2_ electrolysis, (5) bio-feedstock prices, and (6) MPW import emissions.

### Greenfield and brownfield transition strategies

**Figure 5:**
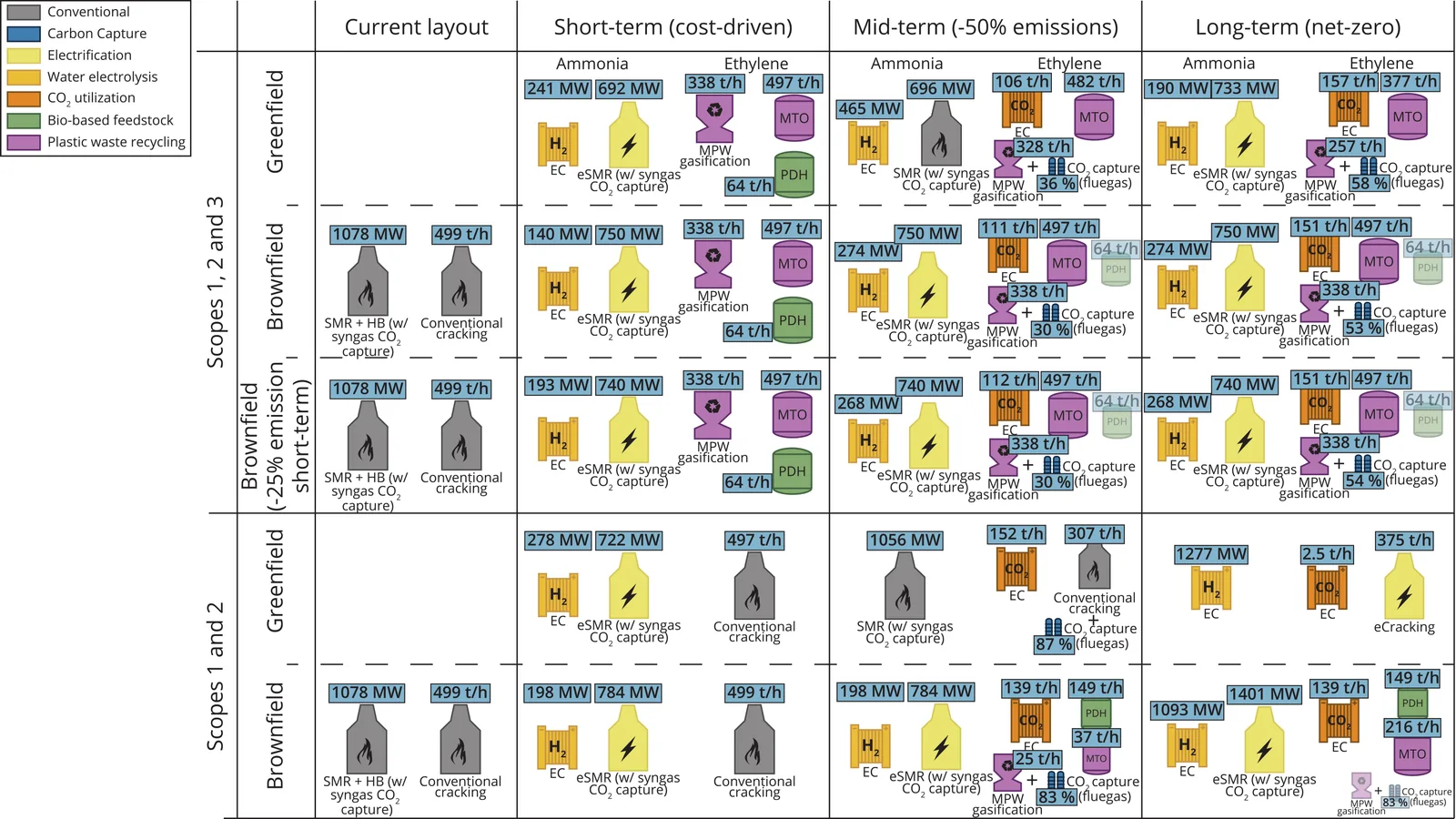
Technology pathways for the baseline scenarios (greenfield and brownfield), with and without scope 3 emissions, for 2030, 2040, and 2050 The technologies shown with lower opacity represent legacy assets in the brownfield scenario that are retained but not operated.

The baseline scenario evaluates transition pathways toward net-zero by 2050, with an interim target of 50% reduction by 2040 compared with 2030 brownfield levels. Figure 5 shows the optimal technology mixes for greenfield and brownfield configurations in 2030, 2040, and 2050, including the scenario excluding scope 3 emissions. Installed capacities of technologies and import/export flows are detailed in Tables S5–S7 in the SI. It is convenient to analyze results moving across the different optimization periods.

#### Short term

**Figure 6:**
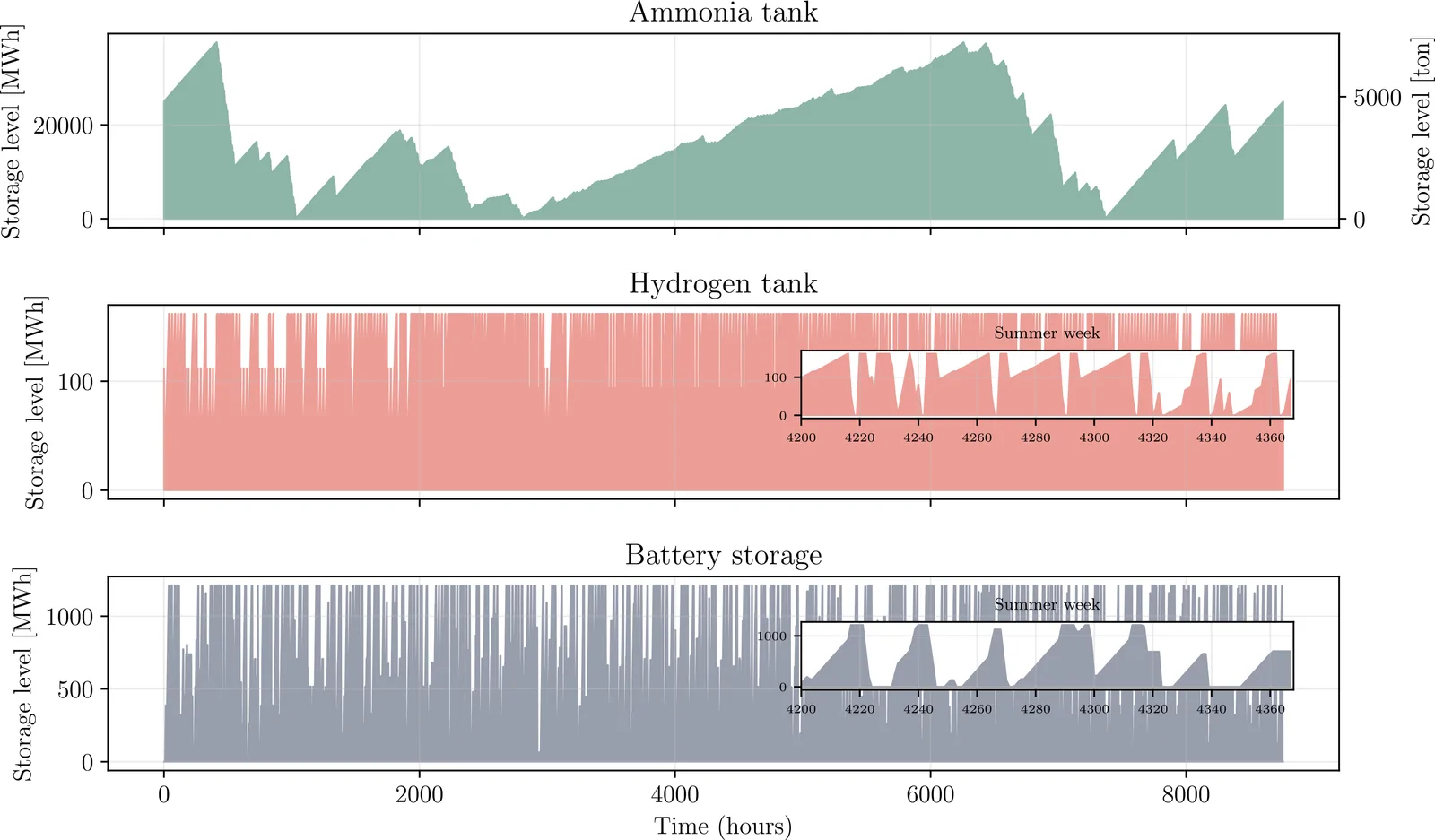
Storage levels of ammonia (top), hydrogen (middle), and battery storage (bottom) in the short-term greenfield scenario throughout the year Each graph also shows a zoom-in in the top right corner with daily operation.

For the short term, results (Figure 5, top two rows) show that greenfield and brownfield scenarios follow similar optimal pathways, with only minor differences in ammonia production capacities. Notably, even though existing assets in the brownfield case could meet demand, the model decommissions and replaces them with newer technologies offering better efficiency, lower emissions, and reduced system costs. More specifically, in ammonia production, conventional SMR is replaced by a combination of Electric Steam Methane Reforming (eSMR) and AEC. While conventional SMR is slightly more energy efficient, eSMR consumes less feedstock, reducing scope 3 emissions, and offers lower Capital Expenditures (CAPEX) per ton of hydrogen. AEC, despite being less efficient and more expensive, runs on electricity alone and provides flexibility during periods of low electricity prices and emissions. In the brownfield case, flexibility in syngas production is constrained by the capacity of the existing HB unit. Although the HB can be expanded, doing so is costly, making it more economical to operate the existing unit at a relatively steady rate, unlike in the greenfield scenario, where the optimal capacity of conventional processes can be optimized as well. Therefore, the more cost-effective strategy is to provide flexibility via storage and a smaller, more flexible AEC, while the eSMR and the HB are operated at constant load. Figure 6 shows that ammonia storage is used seasonally, while hydrogen and battery storage operate diurnally in both scenarios.

For ethylene, the cost-optimal route is MPW gasification to methanol with MTO; this is due to low feedstock costs and minimal external energy needs. The capacity of this route is limited by a 70% recycling cap on MPW imports. Therefore, PDH is chosen to complement the remaining production despite its high CAPEX, as bio-propane is relatively cheap and the process yields valuable hydrogen. These technologies all operate at full load due to their low electricity use, making flexible operation redundant.

#### Mid term

In the mid term, notable differences start to emerge between the greenfield and brownfield scenarios. While both benefit from expanded grid capacity that allows for increased deployment of electrolyzers, grid limitations persist and continue to shape the optimal technology mix and operational strategies. Despite differences in technology choices and operational strategies, mid-term system costs remain closely aligned across scenarios: greenfield costs are 5.5% lower, even after accounting for sunk brownfield investments. This provides two key insights: legacy constraints do not necessarily undermine cost-effectiveness, and multiple near-optimal transition pathways exist, shaped by trade-offs among feedstock and capital costs, emissions, and infrastructure limitations. Notably, the electricity grid limitation is an important active constraint in both the green and brownfield cases, which affects the optimal choice of technologies for ammonia synthesis and which leads to a different cluster configuration depending on the scenario. Due to the high electricity demand of H_2_ and CO_2_ electrolysis (11 MWh/t CO_2_), the greenfield system favors conventional SMR over electric SMR to avoid importing electricity at high lost load prices (Figure 2B), which would occur due to the inflexible operation of the eSMR. Although conventional SMR increases methane use and therefore scope 3 emissions, it enables electric boilers and electrolyzers elsewhere to operate, resulting in overall lower methane imports. However, this difference is not sufficient to justify this change in the brownfield case, where the existing aceSMR from the short-term optimization is kept. When looking at ethylene, the differences between the green and brownfield cases reduce to different optimal sizes. In the brownfield case, PDH remains from earlier investment but is no longer used, while it is not installed in the greenfield scenario. In both scenarios, PDH is replaced by CO_2_ electrolysis, which becomes attractive despite high capital and energy intensity, due to the availability of CO_2_ feedstock within the cluster. MPW gasification remains part of the ethylene supply in both cases. In the greenfield system, smaller new units are installed; in the brownfield case, existing ones operate less frequently. Notably, in the mid term, MPW gasification is equipped with fluegas CC, which reduces its process emissions of about 30–36% in both green-/brownfield cases. The capture rate is limited by the availability of the CO_2_ transport infrastructure, which constrains further reductions despite suitable point-source emissions.

#### Long term

In the long term, both scenarios converge to similar production routes, with different technology sizes remaining as the main deviation. In the brownfield case, sizing is affected by earlier decisions, most notably the oversized storage and redundant PDH. Flexibility and storage become redundant as electricity-related scope 2 emissions are eliminated, indicating that current projections of price fluctuations alone are insufficient to incentivize flexibility. Ammonia production with the hybrid eSMR-AEC setup is chosen in both scenarios, reinforcing it as a low-regret investment. The greenfield pathway, optimized from scratch, yields only slightly different technology choices than the brownfield system, which incurs 12% higher costs due to legacy constraints. While this cost difference is non-negligible, it falls within the plausible uncertainty range in technology and feedstock prices. The expanded 2,500 MW grid connection enables a significant scale-up of CO_2_ electrolysis, especially in the greenfield scenario. This reduces reliance on MPW-MTO pathways there, though these persist at partial load in the brownfield case due to earlier investments. Nevertheless, the MPW-based pathway continues to play a central role in olefin production, as grid limitations and the high electricity intensity of the CO_2_ electrolyzer restrict its ability to fully replace other feedstocks. Moreover, we find that reaching net-zero emissions does not coincide with net-zero fossil fuels: methane remains part of the optimal system, especially for eSMR, due to its lower cost relative to biomethane (2.5 times cheaper) and modest emissions (0.2 ton CO_2_/MWh). In fact, it is possible to remove these emissions by capturing and storing CO_2_ from syngas in the ammonia synthesis and from non-fossil CO_2_ emissions in the MPW gasification. The latter offset is needed to abate the CO_2_ slipping the syngas capture. However, this strategy does not work when dealing with fossil naphtha, as the high carbon content prevents using any of the previous strategies. Additionally, while the current system configuration is sufficient to meet net-zero targets, further emission reductions, or even negative emissions, could be enabled by expanding CO_2_ transport capacity, increasing grid availability, or reducing the cost of bio-feedstocks. Finally, in a net-zero system, the downstream demand for CO_2_ and the CO_2_ input for the electrolyzer must be met with emission-neutral sources. In our model, this is predominantly captured CO_2_ from MPW gasification. The viability of this pathway largely depends on the assumed origin of the waste feedstock. In our base case, we assume that the plastic in the MPW stream is either bio-based or synthetic, which avoids scope 3 emission penalties. Consequently, flue gas CO_2_ from gasification is eligible for capture and reuse. If, instead, fossil-based waste is assumed, the design could shift toward alternative feedstocks, as explored below.

**Figure 7:**
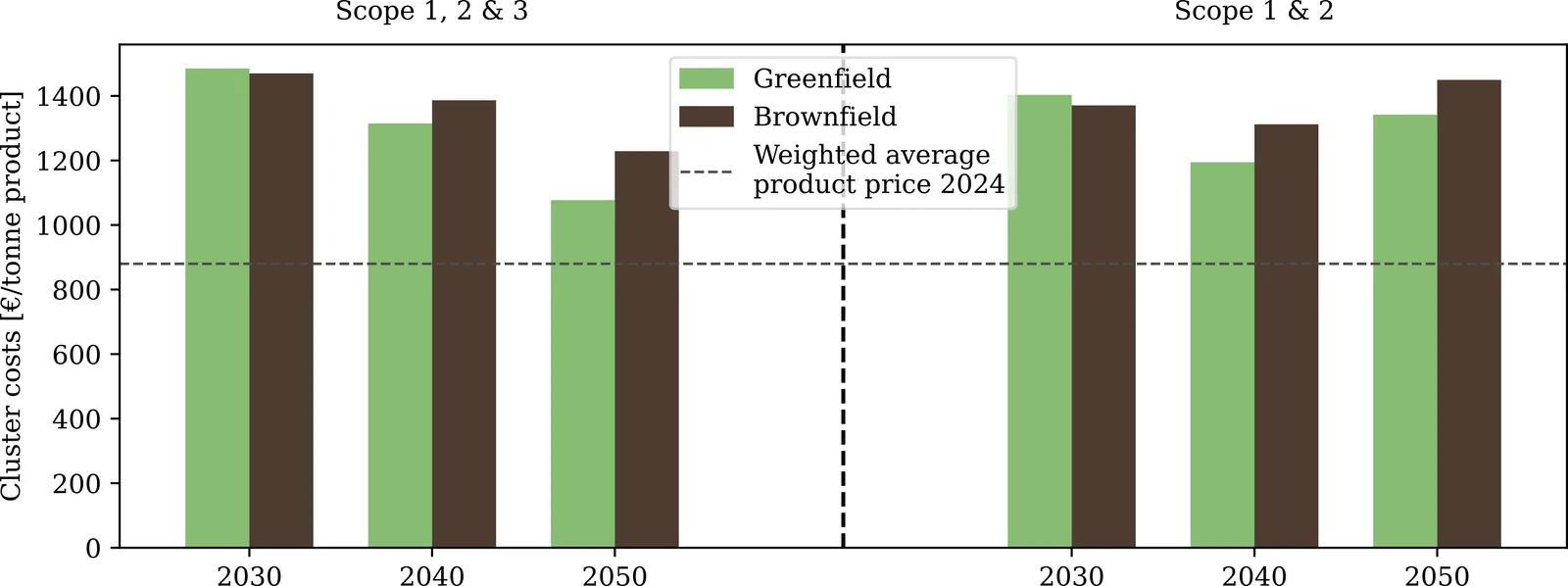
Cluster production costs (€/ton) for greenfield and brownfield configurations in 2030, 2040, and 2050, under full (scope 1–3, left) and energy-only (scope 1–2, right) emission constraints The dashed line indicates the 2024 weighted average market price in Chemelot (€880/ton) for ethylene, propylene, and ammonia.

Figure 7 presents the cluster production costs for ethylene, propylene, and ammonia. Costs range from €1,077 to €1,485 per ton, which is 22%–69% above the 2024 weighted market benchmark of the three key products (€880/ton, see calculation in SI).^54^^,^^55^^,^^56^ While the cost increase is significant, especially in light of the tight profit margins for the sector, these increases reflect the scale of system change needed to reach net-zero emissions. However, it is important to note that cost increases in basic chemicals, which are frequently intermediate products in the production of complex, higher-value goods, do not necessarily result in proportional increases in the final cost of consumer products, as, for example, shown in the case of green steel.^57^

When comparing the cost of brownfield and greenfield, a few messages emerge. In 2030, brownfield systems benefit from existing infrastructure and show slightly lower costs than greenfield systems. However, this advantage reverses in 2040 and 2050 due to lock-in effects: early investments in transitional technologies lead to higher costs as they become obsolete. Cost differences between greenfield and brownfield configurations range from 5.5% to 9.9% in 2040 and 8.0%–14.1% in 2050, but remain within typical uncertainty margins for system cost projections. The lowest system cost occurs for the greenfield net-zero case in 2050, which is driven by declining feedstock costs and reduced need for flexibility measures due to a constant emission-free electricity supply. Conversely, excluding scope 3 emissions leads to higher long-term costs due to continued use of fossil feedstocks and delayed system transformation.

#### Effect of tighter emission limit in the short term

To assess the sensitivity of results on the interim climate policy, we re-optimized the brownfield case with a 25% tighter short-term emission limit (vs. the unconstrained + carbon price 2030 case). The optimal technology mix is shown in the third row of Figure 5. The resulting cluster production costs are €1,476, €1,393, and €1,236 per ton in 2030, 2040, and 2050, respectively. Despite the tighter emission constraint, cluster production costs increase by only 0.4, 0.4, and 0.6% compared with the brownfield case without an emission limit in the short term, well within the 1% MIP gap, due to a trade-off between higher capital costs and lower CO_2_ tax payments. The overall technology mix remains largely unchanged, with key changes limited to larger H_2_ electrolyzer and storage capacities. This suggests that near-term emission reductions can be achieved through added flexibility rather than a shift in core technologies. The consistency of the optimal pathways under tighter constraints reinforces the case for early investment in alternative pathways today, even under uncertainty, rather than delaying action in anticipation of better options emerging later.

#### Effect of scope 3 emissions

As expected, scope 3 emissions play a key role in the transition of chemical clusters: indeed, when optimizing for scope 1 and 2 emissions exclusively, the optimal set of technologies varies significantly in all periods compared with the full emission reduction. Results are illustrated in the bottom two rows of Figure 5.

In the short term, without scope 3 emissions, fossil-based feedstocks remain more attractive, and conventional technologies dominate the optimal portfolio, particularly for olefin production. Ammonia production is the only exception, where the hybrid eSMR-AEC pathway remains cost-effective, confirming that the hybrid pathway is a no-regret investment decision. The preference for conventional fossil technologies also affects the utilization of critical infrastructure, as the electricity grid reaches its maximum capacity only during periods of low electricity prices and low grid emissions to charge the battery, while the average baseload is reduced by a 3- to 4-fold compared with the baseline scenario. This is a direct consequence of not using electrified processes. In contrast, CO_2_ transport and storage becomes the new bottleneck for emission reduction, limiting the amount of CO_2_ that can be sent to underground storage. This highlights again how the adoption of alternative or electrified technologies is tightly coupled with infrastructure availability, underlining the central role of grid capacity for low-emission clusters.

In the mid term, conventional technologies remain prevalent, supplemented by post-combustion capture and utilization in the CO_2_ electrolyzers. However, grid limits constrain electrolyzer operation, preventing full utilization of the captured carbon and resulting in a net export of CO_2_. Similar to when scope 3 emissions are included, the differences between the greenfield and brownfield cases become more pronounced in the mid term. In the greenfield configuration, a hybrid olefin production pathway emerges as optimal, combining conventional naphtha cracking with CO_2_ electrolysis. This configuration reflects the trade-off between technology performance and infrastructure constraints: while CO_2_ electrolysis is preferred from an emissions standpoint, its high electricity demand cannot be fully accommodated due to limited grid capacity. Consequently, only partial deployment is feasible. In contrast, the brownfield scenario is subject to stricter design constraints due to the presence of existing assets. Because part-load operation or partial decommissioning of the steam cracker is not allowed, the model opts to fully decommission the unit and replace it with alternative technologies such as CO_2_ electrolysis, PDH and MPW gasification. As a result, emissions are reduced by 56%, exceeding the 50% target. This highlights again how legacy infrastructure and retrofit constraints can significantly shape emission reduction pathways.

The effect of excluding scope 3 emissions becomes particularly strong in the long term. While fossil feedstocks remain part of the optimal solution in all cases, the mix shifts: methane is favored when scope 3 emissions are included, whereas accounting for only scope 1 and 2 leads to a greater role for more carbon-intensive naphtha. In the greenfield case, H_2_ and CO_2_ electrolysis and electric naphtha cracking are deployed. In the brownfield case, the eSMR capacity is expanded to supply syngas for olefin production. However, legacy assets and infrastructure constraints restrict CO_2_ electrolysis to a quarter of its capacity and eliminate MPW gasification operation. This underutilization reflects how legacy assets can limit the deployment of preferred technologies. Notably, the impact on cluster production costs is rather pronounced, with an increase of 13.5%.

Interestingly, the cost trajectories of the case with and without scope 3 emissions diverge. The scope 3-inclusive pathway sees declining levelized costs due to early investment in scalable, low-emission technologies. In contrast, the scope 1–2 case incurs rising costs by 2050 due to early lock-in, which requires expensive replacements later. This highlights how including scope 3 emissions can guide more robust and cost-effective long-term transitions.

### Zeeland case study

**Figure 8:**
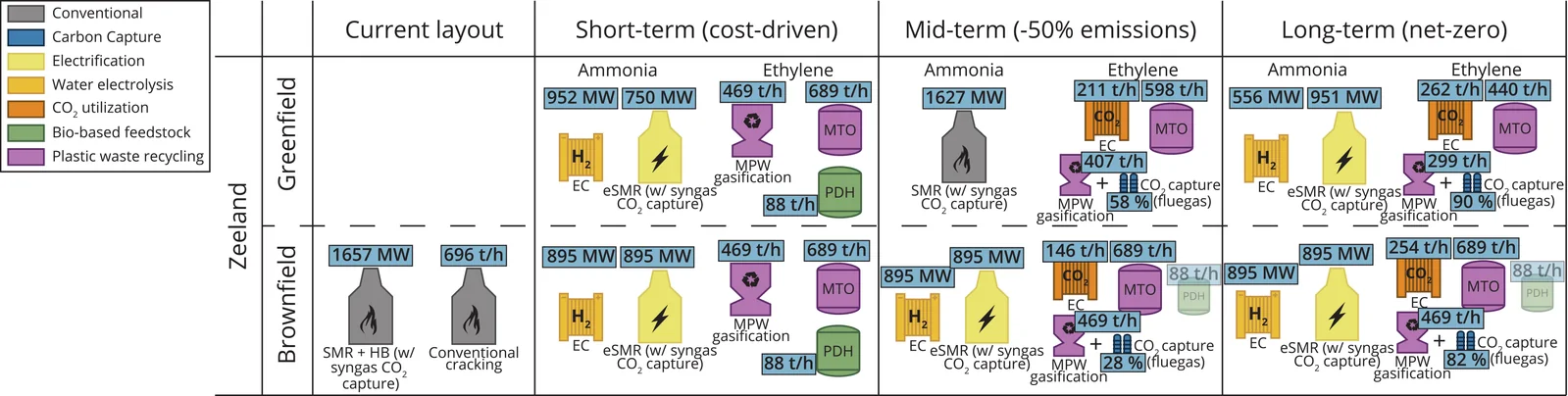
Technology pathways for the scenarios including scope 3 emissions (greenfield and brownfield) over the short-term (2030), mid-term (2040), and long-term (2050) for the Zeeland cluster

**Figure 9:**
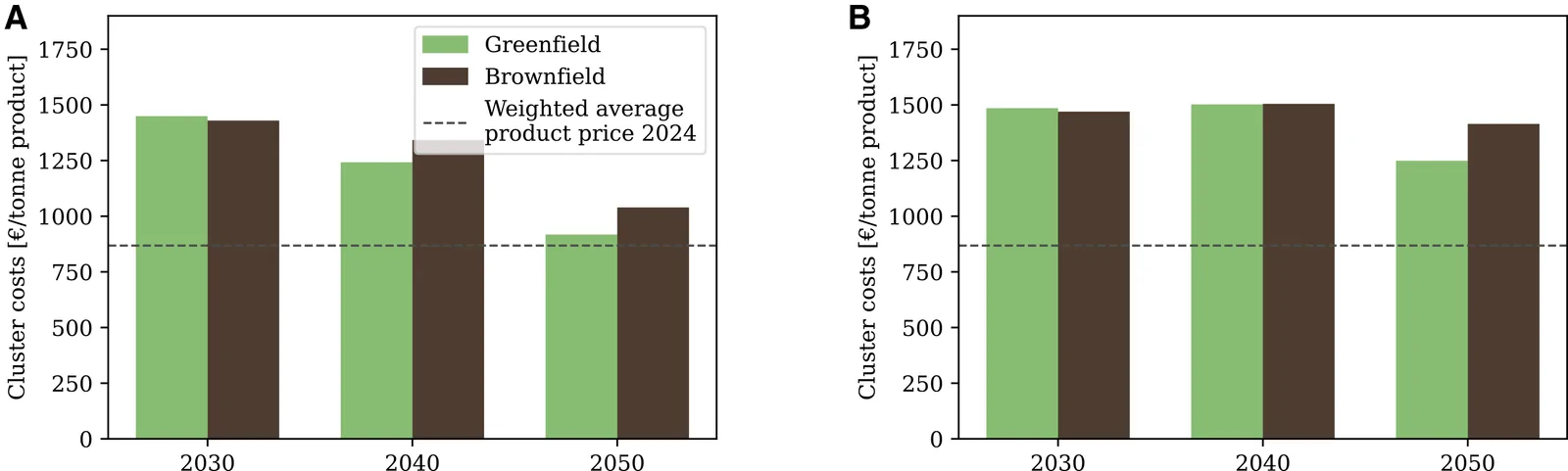
Cluster production costs (€/ton product) for Greenfield and Brownfield configurations across 2030, 2040, and 2050, for the Zeeland cluster and the Chemelot cluster without CO_2_ electrolysis The horizontal dashed line indicates the weighted average product price (of ethylene, propylene, and ammonia) for Zeeland (868 €/ton) in A, and for Chemelot (880 €/ton) in B, serving as a benchmark for economic feasibility. (A) Zeeland. (B) No CO_2_ electrolyzers.

The results for Chemelot suggest that infrastructure constraints, particularly the electricity grid and CO_2_ transport capacity, can strongly influence transition pathways. To assess the impact of improved infrastructure, we analyze the case of Zeeland, which benefits from a larger grid connection and greater CO_2_ transport capacity due to its strategic location. We re-run both greenfield and brownfield optimizations, including scope 3 emissions. Figure 8 shows the resulting pathways; full technology capacities and import/export levels are listed in Tables S5 and S9 in the SI. The model again identifies the same optimal technologies as at Chemelot, AEC, and eSMR for ammonia, and MPW gasification, MTO, and CO_2_ electrolysis for ethylene, indicating the robustness of these configurations across regions. However, Zeeland’s enhanced infrastructure enables key improvements: higher-capacity electrolyzers can be installed and operated, and expanded CO_2_ infrastructure supports greater capture and utilization. These advantages translate to lower cluster production costs (Figure 9A), particularly in 2050. This underscores how infrastructure availability and geographic context critically shape both the feasibility and economics of low-emission industrial transitions, reinforcing the value of spatially explicit, cluster-level models.

### No CO_2_ electrolyzers

Results have shown that CO_2_ electrolysis plays a key role in enabling a cost-effective transition in the mid and long term. Indeed, it allows for valorizing the captured CO_2_, a nearly cost-free by-product of other processes. However, this technology has a lower readiness level compared with the others considered in this study, and questions remain whether it will be commercially viable by 2040 as assumed. To assess the implications of delayed or unavailable deployment, we test the case of excluding CO_2_ electrolysis from the technology portfolio. As summarized in Tables S6 and S9 in the SI, its exclusion leads to increased use of alternative technologies already favored in the baseline, particularly PDH and MPW gasification. Interestingly, the system leans more heavily on bio-based carbon sources (e.g., biomass and biogenic plastics) rather than other CO_2_ utilization pathways, such as the electricity-intensive methanol-based routes, which overall require 70%–75% more electricity per ton of olefins than CO_2_ electrolysis. The shift in feedstock also affects the ammonia production pathway: in the absence of CO_2_ electrolysis, AEC is preferred over eSMR, as CO_2_ in the syngas cannot be converted. These changes highlight the interdependencies between olefin and ammonia systems and demonstrate the value of integrated cluster-level analysis. Finally, as shown in Figure 9B, the exclusion of CO_2_ electrolysis leads to 8.5%–16% higher cluster production costs in 2040 and 2050, confirming its role in enabling a more cost-effective transition. These findings emphasize the strategic value of supporting the development and deployment of CO_2_ electrolysis technologies, particularly in regions with adequate grid capacity and abundant renewable generation potential. In parallel, planning efforts should account for robust alternatives such as biomass and recycled plastic feedstocks, which can become more favorable in the absence of CO_2_ electrolysis and/or access to low-price biomass.

### Optimistic bio-feedstock prices

**Figure 10:**
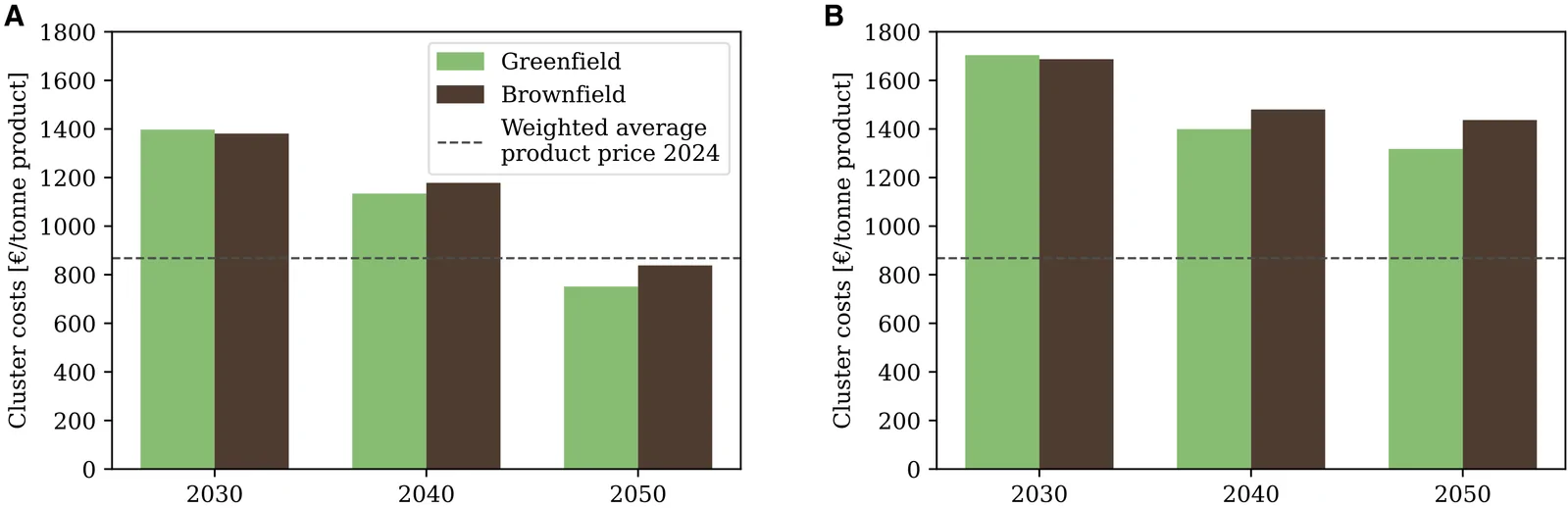
Cluster production costs (€/ton product) for Greenfield and Brownfield configurations across 2030, 2040, and 2050, for the sensitivity analyses in the Chemelot cluster with optimistic bio-feedstock prices and with an MPW emission factor The horizontal dashed line indicates the weighted average product price (of ethylene, propylene, and ammonia) for Chemelot (880 €/ton), serving as a benchmark for economic feasibility. (A) Optimistic Bio-Feedstock Prices. (B) Scope 3 Emissions from fossil MPW.

In the baseline net-zero case, fossil methane is still adopted for its relatively low scope 3 emissions and the relatively simple offset through CO_2_ capture and storage. While bio-based feedstocks offer an alternative to fossil feedstocks, their high prices (i.e., limited availability) limit deployment. To assess the impact of assumptions on future prices, which are often proven wrong in reality, we tested a scenario with significantly reduced bio-feedstock prices (between brackets in Table 2). Not surprisingly, the results show a clear shift toward bio-based pathways. Cheaper bio-propane makes PDH the preferred olefin route, supplemented by MPW gasification in 2030 and CO_2_ electrolysis from 2040 onwards. The hydrogen by-product from PDH removes the need for dedicated hydrogen production via AEC, and the new configuration lowers demand for grid electricity and CO_2_ transport/storage, highlighting the benefits of bio-feedstocks in integrated system optimization. Technologies with feedstock flexibility, such as eSMR, remain robust. Initially running on fossil methane, eSMR switches to bio-methane from 2040. This highlights the strategic value of feedstock-flexible technologies that can adapt to evolving market and policy conditions. As shown in Figure 10A, reduced bio-feedstock prices lead to deeper emission reductions and lower system costs. In the greenfield case, system costs are minimized at 92% emission reduction by 2040, far exceeding the required 50% cut relative to 2030, while the brownfield scenario reaches net-negative emissions. The cluster production costs drop 2%–30% below the baseline and fall beneath the 2024 weighted average product price by 2050. These findings illustrate the transformative potential of affordable bio-feedstocks but also emphasize the uncertainty surrounding their long-term availability and pricing, making technology feedstock flexibility a key asset in low-regret transition planning.

### Fossil MPW emission rate

Accounting for scope 1, 2, and 3 emissions from MPW gasification is challenging, as it depends heavily on the origin of the plastic waste, whether fossil-based, bio-based, or synthetic. At the industrial cluster level, this information is often unavailable, requiring assumptions about the feedstock composition and systemic losses. In our baseline scenario, we assume that MPW is net-zero in emissions, implying that both the original plastic and any carbon losses during collection, sorting, and gasification are balanced by net-zero plastic sources. While this assumption may be valid in the long term, it is likely overly optimistic for the short term, when fossil-based plastics still dominate the waste stream. To test a more conservative case, we re-optimize assuming MPW is entirely fossil-based, assigning a scope 3 emission factor of 2.38 tons CO_2_ per ton of MPW. This adjustment has profound consequences: MPW gasification is partially replaced by PDH in the short term and CO_2_ electrolysis in later decades. As with earlier scenarios, hydrogen co-produced via PDH removes the need for standalone AEC. Emissions increase 7-fold versus the baseline, not only due to the scope 3 emissions from imported MPW, but also because the flue gas CO_2_ from MPW gasification no longer qualifies as carbon neutral. As a result, it cannot be used as a neutral carbon source for downstream demand or olefin production. These higher emissions and increased reliance on more expensive alternative feedstocks, like bio-propane, raise the cluster production costs by 6%–22% relative to the baseline (Figure 10B). Despite this, the system design still favors fossil MPW over switching to other bio-feedstocks in the short term, underlining its cost and energy efficiency advantages for olefin production. This scenario underscores the strategic but emissions-sensitive role of MPW gasification and highlights the importance of transparent, standardized scope 3 accounting in industrial transition modeling.

### Summary and brownfield transition pathways

Our baseline results show that while brownfield configurations lead to slightly different system designs and higher costs, core technology choices remain consistent across both cluster types and time horizons. This consistency indicates the presence of low-regret options and supports early investment rather than deferring decisions in the hope of improved future uncertainty.

**Figure 11:**
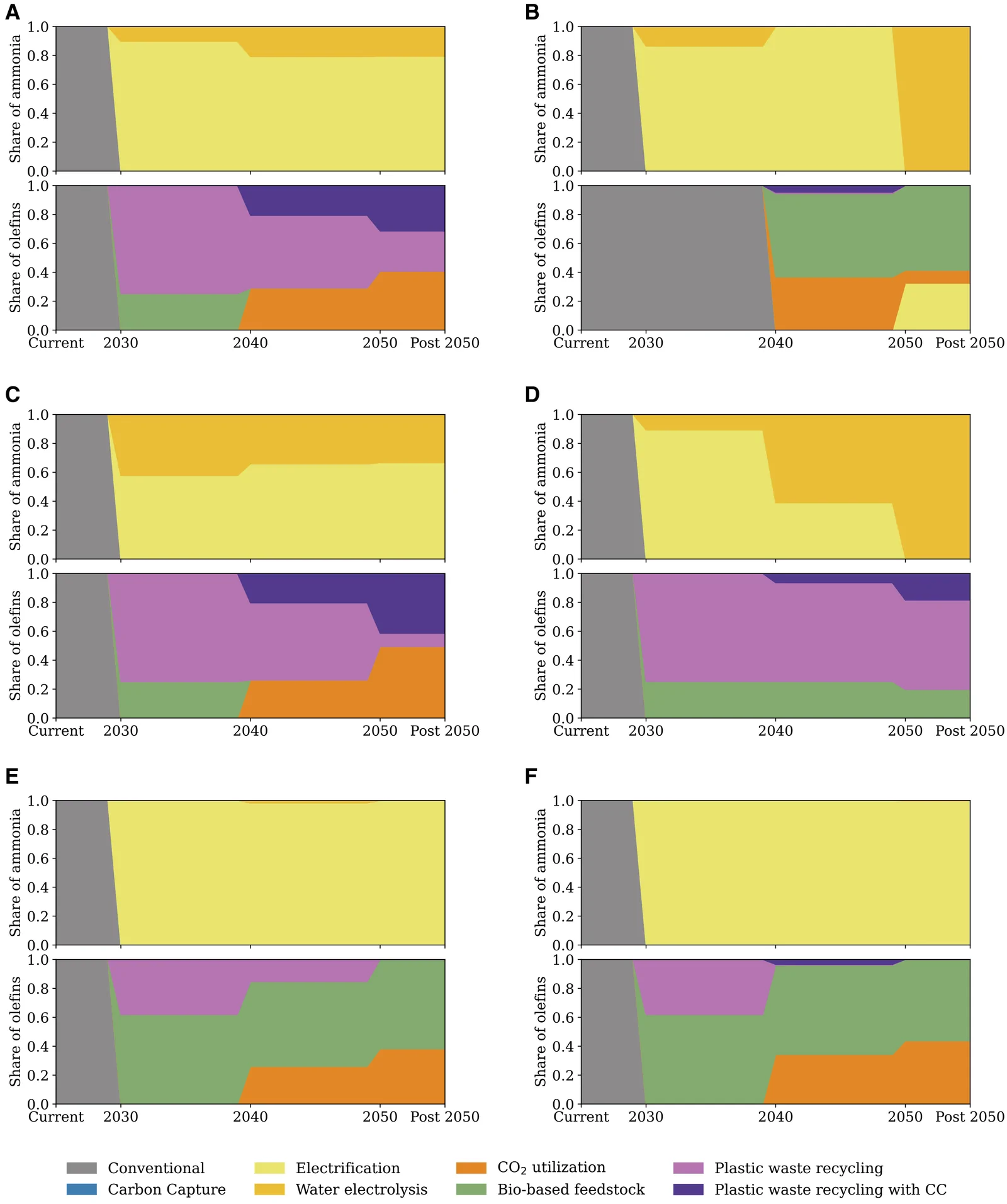
Optimal brownfield transition pathways for olefins and ammonia under the baseline and sensitivity scenarios Colored areas indicate the annual production shares of ammonia (top) and olefins (bottom) by technology type, relative to total production in each year. 2030, 2040, and 2050 are investment decision moments when the technology portfolio is re-evaluated. (A) Chemelot: scope 1, 2, and 3. (B) Chemelot: scope 1 and 2. (C) Zeeland (higher CO_2_ transport capacity and electricity connection). (D) No CO_2_ electrolyzers available. (E) Optimistic Bio-Feedstock Prices. (F) Fossil MPW Emission Rate.

The transition pathways are summarized in Figure 11, which illustrates how emissions accounting and infrastructure availability strongly shape the brownfield cluster transformation. In the short and mid term, excluding scope 3 emissions (Figure 11B) encourages reliance on fossil-based technologies, delaying electrification and leading to costly reinvestments after 2040 to meet net-zero scope 1–2 targets. In contrast, full-scope accounting supports a more consistent and scalable deployment of low-carbon technologies, lowering cumulative system costs. Regional infrastructure access also plays a pivotal role: for instance, the Zeeland case (Figure 11C) benefits from greater grid capacity and CO_2_ transport and storage infrastructure, enabling more electrification and carbon utilization through CO_2_ electrolysis.

Sensitivity analyses reveal that the exact technology mix and the corresponding system costs and emissions are highly sensitive to boundary conditions. Excluding CO_2_ electrolysis shifts the system toward near-optimal technologies like PDH and MPW gasification, rather than alternative CO_2_ utilization pathways. Lower bio-feedstock prices (Figure 11E) enable cheaper, bio-based pathways with CO_2_ utilization. In contrast, assuming fossil-based MPW (Figure 11F) increases emissions 7-fold and raises costs significantly. These findings underscore the need for robust transition planning that accounts for uncertainty and its system-wide effects. While all scenarios achieve net-zero by 2050, the path taken and the cost of getting there depend strongly on external assumptions.

## Discussion and limitations

This study offers new insights into how chemical clusters can transition toward net-zero emissions in a cost-effective manner. The proposed framework captures the complex trade-offs and lock-in risks inherent in long-term planning by comparing greenfield and brownfield transitions, incorporating several detailed representations of technologies, feedstock choices, infrastructure, and emissions scopes. The analysis reveals that while greenfield developments offer more design flexibility, brownfield configurations can achieve net-zero while offering opportunities to reuse existing infrastructure. Importantly, we find that even under varying conditions and uncertainties, certain technologies and system designs consistently emerge as low-regret options, supporting early action over strategic delay.

As is often the case in modeling, this study features certain limitations. First, the model focuses on production routes for ammonia and olefins, excluding downstream products and co-processing streams. These additional products can significantly influence the cluster’s material integration and cost structure (as shown in Tan et al.^11^ and Manalal et al^58^), potentially leading to different system designs and unrealized system integration opportunities. Second, the model relies on deterministic inputs and projections for emerging technologies. This is also reflected in the limited representation of long-term dynamics: here, we consider the 2030–2050 evolution of electricity price and carbon intensity, but we do not consider, for example, market, demand, and resources variations. While we address uncertainties with sensitivity analyses, the deterministic nature of the analysis means that the conclusions drawn are conditional on the selected scenarios and input assumptions, and should be interpreted as scenario-specific insights rather than universally generalizable results. Future work could apply stochastic optimization to fully embed uncertainties in future conditions in the analysis. For this to happen, however, progress on algorithms and methods is needed to cope with computational complexity. Third, while this study includes a broad range of alternative pathways for chemical production, some promising technologies are excluded, for example, biomass gasification. This was necessary to keep the problem tractable from a computational perspective. We, however, note that the open-access model we use is designed for straightforward integration of additional technologies that might be of interest for specific readers. Along this line, we note that the transitions identified in this work are only possible if the considered technologies become commercial by the adoption time; this is subject to additional uncertainty, as failing to commercialize would require a different solution (we test this for CO_2_ electrolysis). Fourth, emissions accounting critically shapes outcomes; for example, classifying MPW as fossil-based versus biogenic/synthetic shifts technology needs from carbon-neutral to carbon-removal strategies. Fifth, higher-level impacts like land use, supply and distribution chain emissions, and market interactions, including competition for limited bio-feedstocks or opportunities for shared infrastructure are also outside the scope. In particular, we note that biomass availability at cluster level strongly depends on national strategies (both at company and at policy makers’ strategic level); for example, biomass might be unavailable should its use be prioritized for non-chemical applications. This would shape the optimal technology mix accordingly. Including systemic interactions and linking clusters to national models would offer further insights into regional transition dynamics.

Finally, this study does not consider several aspects of real-world investment behavior and project execution, including construction times, site inactivity during retrofits, firm-specific return-on-investment requirements, detailed financing structures, site space availability and local supply chains, and legislative and regulatory constraints. It is important to emphasize that these financial and practical considerations may ultimately dominate real-world decision outcomes, potentially leading to investment choices that deviate significantly from the cost-optimal pathways identified here. These limitations should be considered when interpreting the results. Complementary approaches, such as detailed techno-economic case studies, financial modeling, agent-based simulations, or stochastic analyses, are better suited to address these dimensions and represent possible avenues for future work building on the insights provided here.

Building on these limitations, the following research directions emerge. First, extending the model to include downstream and national scales could improve infrastructure planning and reveal synergies or showstoppers. A hybrid approach, linking detailed cluster models with broader-scale ones, could improve scalability while ensuring case study comparability. Second, more systematic treatment of uncertainties would help assess how well transition strategies perform under a range of plausible futures, supporting more resilient decision-making. Third, inclusion of real-world investment behavior and project execution in the MILP framework would provide additional insights for decision-makers. Finally, representing investment decisions at a yearly temporal granularity, rather than through multi-year periods, could better reflect real-world decision-making: this would require new methods to preserve operational resolution and computational tractability.

### Concluding remarks

This study presents a comprehensive MILP-based framework to evaluate transition pathways for chemical clusters, integrating current, commercial, and advanced conversion technologies, feedstock switching, and scope 1–3 emissions accounting. Moreover, we coupled rigorous MILP optimization to brownfield, multi-period transition analysis for the chemical industry, therefore improving how real decision-making processes are represented in modeling studies. We applied this framework to two real-world Dutch chemical clusters, comparing greenfield and brownfield configurations under varying emissions targets, infrastructure constraints, feedstock, and technological assumptions.

We found that several near-term investments, such as eSMR with AEC for ammonia and MPW gasification with MTO for olefins, consistently emerge as cost-optimal across scenarios. Their robustness highlights the presence of low-regret options that can guide early decisions despite long-term uncertainty.

Second, we found that legacy assets significantly shape brownfield transition strategies. Although both greenfield and brownfield configurations converge toward similar 2050 portfolios, early investments can create path dependencies, raising long-term costs by up to 14% compared with greenfield systems. Including downstream scope 3 emissions in early-stage targets (e.g., 2030) helps mitigate the risk of lock-in, as it encourages early investment in future-proof technologies.

Third, infrastructure availability, particularly electricity grid capacity and CO_2_ transport, emerged as a key determinant of the system’s ability to reduce emissions. Additionally, our analysis emphasizes the importance of operational flexibility in electrification pathways, especially in light of high uncertainty about future energy system dynamics. As shown in the Zeeland case study, improved infrastructure enables more electrification and CO_2_ utilization, reducing reliance on fossil inputs and enabling a more cost-effective transition to net-zero chemical production.

Finally, transition pathways are highly sensitive to uncertain parameters, including technology availability, bio-feedstock prices, and waste classification. This highlights the need to test the performance of fixed system designs under uncertain external conditions. Doing so will strengthen the credibility of model insights and support the development of resilient, adaptable transition strategies.

Overall, our findings imply that coordinated infrastructure planning and early investment in low-regret, full-scope mitigation technologies are essential. Multiple near-optimal pathways exist, shaped by interdependent trade-offs among feedstock availability, capital costs, emissions intensity, and infrastructure limitations. While future conditions cannot be predicted with certainty, incorporating realistic assumptions and likely scenarios into the modeling process is essential for guiding effective decision-making. Moreover, while this uncertainty remains, delaying action in anticipation of perfect foresight is unlikely to yield substantially better outcomes. Instead, prioritizing robust, flexible technologies and enabling infrastructure today can accelerate the transition to net-zero chemical clusters in a cost-effective and prudent manner.

## Resource availability

### Lead contact

Further information and requests for resources should be directed to and will be fulfilled by the lead contact, Matteo Gazzani (m.gazzani@uu.nl).

### Materials availability

This study did not generate new unique reagents or materials.

### Data and code availability

• The data supporting this study, including additional technical details, cost and performance data for chemical processes and storage technologies, weighted average market price calculations, design day selection, and sensitivity analysis results, are available in the supplemental information. The file Electricity_data.xlsx, containing hourly electricity prices and grid emission rates for the Chemelot and Zeeland case studies, is provided with the article. Additional input datasets and raw model outputs are available in the public repository archived at https://doi.org/10.5281/zenodo.15974378.
• The modified version of the AdOpT-NET0 framework used in this study, together with the scripts required to reproduce the analyses, is publicly available at https://github.com/julia1071/AdOpT-NET0_ClusterNet0_paper and archived at https://doi.org/10.5281/zenodo.15974378. The repository is released under the MIT License.
• Any additional information required to reproduce the results reported in this paper is available from the lead contact upon reasonable request.

## Acknowledgments

This research was partly funded by 10.13039/501100019926TNO (10.13039/501100019926Netherlands Organisation for Applied Scientific Research) as part of the Industrial Transformation framework.

## Author contributions

J.L.T., conceptualization, data curation, formal analysis, investigation, methodology, software, visualization, and writing – original draft; G.J.K., methodology, validation, supervision, and writing – review and editing; A.P.C.F., funding acquisition, supervision, and writing – review and editing; M.G., conceptualization, methodology, validation, visualization, supervision, and writing – review and editing.

## Declaration of interests

The authors declare no competing interests.

## Declaration of generative AI and AI-assisted technologies in the writing process

During the preparation of this work, the author(s) used ChatGPT in order to improve the readability of the text. After using this tool/service, the author(s) reviewed and edited the content as needed and take(s) full responsibility for the content of the published article.

## STAR★Methods

### Key resources table

**Table:** 

REAGENT or RESOURCE	SOURCE	IDENTIFIER
**Deposited data**		

Cost parameters of the conventional and alternative chemical production processes	This paper (individual references specified in the SI)	S1
Performance parameters of conventional and alternative chemical production processes	This paper (individual references specified in the SI)	S2
Annual cluster production capacities	Manufacturing Industry Decarbonisation Data Exchange Network (MIDDEN)	https://www.pbl.nl/en/middenweb/the-database
Hourly electricity prices and carbon rates	In Supplementary Information (from I-ELGAS model, TNO)	Electricity_data.xlsx
Feedstock and energy prices	This paper	Table 2
CO_2_ tax	Dutch Emission Authority (NEA). Tariff was lowered to 78,67 in 2026.	https://www.emissieautoriteit.nl/regelgeving/co2-heffing/tarieven-co2-heffing
CO_2_ transport and storage capacity and costs, electricity grid capacity	This paper	Table 1

**Software and algorithms**		

AdOpt-NET0 optimization framework	Github; Journal of Open Source Software	https://github.com/UU-ER/AdOpT-NET0; https://joss.theoj.org/papers/10.21105/joss.07402

### Method details

The analysis was performed using the open-source optimization framework AdOpT-NET0 (Advanced Optimization Tool for Networks and Energy Technologies).^16^ AdOpT-NET0 is a technology-rich energy system optimization framework that formulates multi-energy systems as linear or mixed-integer linear programming (MILP) problems. The framework represents the investment and operation of conversion, storage, and transport technologies across multiple energy and material carriers, enabling the design of cost-optimal systems subject to technical, economic, and environmental constraints. The software has previously been applied to a range of industrial energy system studies, including chemical cluster optimization.^10^

For this work, AdOpT-NET0 was extended to evaluate long-term transition pathways for existing chemical clusters. Three principal developments were implemented. First, the framework was expanded from single-period optimization to a multi-period formulation representing sequential investment decisions in 2030, 2040, and 2050. Second, a brownfield formulation was introduced, allowing existing industrial assets to be retained, retrofitted, or decommissioned while accounting for the influence of legacy infrastructure on future investment decisions. Third, the technology portfolio was expanded with additional production routes, feedstock options, and emissions accounting to capture scope 1, 2, and 3 greenhouse gas emissions, enabling the assessment of both upstream and downstream emission reduction strategies.

The resulting optimization problem determines the cost-optimal combination of technology investments and hourly operation while satisfying mass and energy balances, technology performance constraints, infrastructure limitations, and emission targets. Although the framework represents technologies and system operation in considerable detail, it does not explicitly model market dynamics or endogenous technology learning. Instead, technology costs, fuel prices, emission factors, and infrastructure assumptions are prescribed for each planning period based on externally defined scenarios.

A complete mathematical formulation of the optimization problem, including decision variables, objective function, and constraints, is provided in the main manuscript and Supplementary Information. The AdOpT-NET0 source code is publicly available,^16^ and all model extensions developed for this study are included in the version associated with this work.

### Quantification and statistical analysis

This study is based on mathematical optimization and scenario analysis rather than experimental measurements. Therefore, no statistical tests, biological replicates, sample size estimation, randomization, or anonymization procedures were applicable.

The optimization model was formulated as a mixed-integer linear programming (MILP) problem and solved using Gurobi Optimizer version 12.0. The model determines cost-optimal technology investments and operational strategies while satisfying technical constraints, emission targets, and system balances. All model runs were performed using deterministic input parameters defined for each scenario.

The robustness of the identified transition pathways was evaluated through sensitivity analyses on key uncertain parameters, including technology availability, bio-feedstock prices, fossil waste emission factors, electricity grid capacity, and CO_2_ transport infrastructure. Results are reported as absolute values or relative changes compared with the reference scenarios, as described in the corresponding figure legends and Results sections.

## Supplemental information

Document S1. Figures S1 and S2, Tables S1–S11, and supplemental methods and references
