# Supplementary material for: Rethinking chemical clusters: Greenfield and brownfield transition to net-zero by 2050
Source: iScience. 2026 Sep 5;29(9):117077. doi: 10.1016/j.isci.2026.117077 (PMC13571977; doi:10.1016/j.isci.2026.117077)
Supplement: Supplementary file 1 [file mmc1.pdf]

iScience, Volume *29*

Supplemental information

Rethinking chemical clusters: Greenfield and brownfield transition to net-zero by 2050

Julia L. Tiggeloven, André P.C. Faaij, Gert Jan Kramer, and Matteo Gazzani

Rethinking Chemical Clusters: Greenfield and Brownfield Transition to Net-Zero by 2050

Julia L. Tiggeloven,[†,‡] André P.C. Faaij,[†,‡] Gert Jan Kramer,[†] and Matteo Gazzani*,[†,¶]

†*Utrecht University, Copernicus Institute of Sustainable Development, Princetonlaan 8a, Utrecht, 3584 CB, Netherlands*

‡*TNO, Energy Transition Studies, P.O. Box 37154, Amsterdam, 1030 CE, Netherlands*

¶*Sustainable Process Engineering Chemical Engineering and Chemistry, Eindhoven University of Technology, Eindhoven, 5612 AP, Netherlands*

E-mail: m.gazzani@uu.nl

List of Abbreviations

AEC Alkaline Electrolysis Cell

ASU Air Separation Unit

CC Carbon Capture

EDH Ethanol Dehydration

eSMR Electric Steam Methane Reforming

HB Haber Bosch

MTO Methanol-to-Olefins

MPW Mixed Plastic Waste

OE Operation Envelope

PDH Propane Dehydrogenation

rWGS reverse Water Gas Shift

WAPP Weighted Average Product Price

WSP Warm Standby Power

List of Mathematical Symbols

**Indices**	
$d \in \mathcal{D}$	Set of decade intervals
$m \in \mathcal{M}$	Set of technologies
$m' \in \mathcal{M}'$	Subset of medium-dynamic technologies
$m'' \in \mathcal{M}''$	Subset of storage technologies
$t \in T$	Set of timesteps
**Variables**	
F	Consumption of technology
g	Binary charging storage technology
L	Storage level
P	Production of technology
S	Technology size
x	Binary on/off status plant
**Parameters**	
η^C, η^D	Charging and discharging efficiencies
ϕ	Warm Standby Power fraction

Additional technology information

This section provides additional technical details on selected chemical conversion processes modeled in the study, including reverse water gas shift (rWGS) to methanol, ethanol dehydration (EDH) to ethylene, and propane dehydrogenation (PDH) to propylene.

In the **rWGS**, syngas is first produced from CO_2 and H_2 via an endothermic reaction at around 850 °C, followed by CO_2 and CO hydrogenation to methanol. The required heat for the rWGS reaction is assumed to be supplied by hydrogen combustion, increasing the process's hydrogen consumption by 0.186 MW/t CO_2. To adjust the syngas to the appropriate CO_2/H_2 ratio for methanol synthesis, additional hydrogen is added after the rWGS reactor. After methanol synthesis, methanol is purified to $<$99.5 % by distillation, which requires low-temperature heat supplied by electric heat pumps.

EDH to ethylene is an endothermic catalytic reaction that can be integrated with a bioethanol plant or operated as a stand-alone facility. Although integrating with a bioethanol plant offers benefits such as heat integration, this study focuses on optimizing net-zero chemical production within existing clusters. Consequently, bioethanol production is considered out of scope. In a stand-alone EDH plant, bioethanol (95 wt %) is liquid and must be vaporized before entering the furnace. Ethanol is mixed with medium-pressure steam and introduced into the reactor at 350 °C. The reactor effluent is then cooled, and the products are separated.

PDH to propylene is a highly endothermic catalytic process typically operated at high temperatures, resulting in significant energy demand. Energy efficiency can be enhanced by separating the hydrogen by-product – e.g., using membrane reactor technology – to shift the thermodynamic equilibrium toward propylene. The reactor setup includes four parallel packed-bed reactors operating at 625 °C and 0.5 bar. The propylene-rich stream is cooled and compressed to 35 bar and -30 °C. Hydrogen produced during the process is partially utilized for heat integration, with the excess available for other applications.

Dynamic operation constraints

Given the diversity of processes and conversion technologies present in chemical clusters, we distinguish between three categories: 1) Slow technologies, 2) Medium-dynamic technologies, and 3) Fast technologies. While all three categories of technologies follow the constraints described in the main text, we have additional considerations per type:

- **Slow technologies:** Conventional production processes typically fall into the category of slow technologies, characterized by small operation envelope (OE)s, slow ramping behavior, and extended start-ups and shut-down times of up to 24 hours or more. To accommodate this, we linearize Equation 12 in the main text by excluding $x_{m,t,d}$ for these technologies (implying no start ups and shut downs), allowing for operation within their OE only.

- **Medium-dynamic technologies:** Medium-dynamic technologies typically have an OE exceeding 50% and allow for start-ups and shut-downs of the production, while maintaining the process at a warm standby by consuming electricity. Depending on the technology, the ramping behavior may be constrained. It is worth noting that, for these technologies, we assume that the maximum load change rate $\frac{S_{m,d}}{RT}$ is greater than or equal to the minimum operating point $\lambda_m S_{m,d}$ to ensure that the technology can go from idle to the minimum operation state within one hour. Additionally, we introduce a constraint to account for warm standby:

$$F_{m',\mathrm{electricity},t,d} \geq (1 - x_{m',t,d})\ \phi_{m'}\ S^{\mathrm{ref}}_{m',d} \qquad \forall \quad m' \in \mathcal{M}', t \in T, d \in \mathcal{D} \tag{1}$$

  Here, $\phi_{m'}$ represents the WSP as a fraction of the maximum electric power of the reference size $S^{\mathrm{ref}}_{m',d}$ of medium-dynamic technology $m' \in \mathcal{M}' \subset \mathcal{M}$. Consequently, the equality sign in Equation 10 in the main text is replaced by an inequality sign.

- **Fast technologies:** With an OE of 100% and unconstrained ramping behavior, fast

technologies can operate without ramping limitations in an hour. Hence, we linearize Equation 12 in the main text by excluding $x_{m,t,d}$ while Equation 13 in the main text is dropped.

Storage operation constraints

The storage technologies incorporate the storage level $L_{m'',t,d}$ as an additional decision variable in their performance model, subject to the following constraint:

$$0 \leq L_{m'',t,d} \leq S_{m'',d} \qquad \forall \quad m'' \in \mathcal{M}'', t \in T, d \in \mathcal{D} \tag{2}$$

Here, $\mathcal{M}''$ represents a subset of storage technologies within the broader technology set $\mathcal{M}$. Furthermore, the charging and discharging behavior is expressed as

$$L_{m'',t,d} = L_{m'',t-1,d} + F_{m'',t,d}\eta^C_{m''} - \frac{P_{m'',t,d}}{\eta^D_{m''}} \qquad \forall \quad m'' \in \mathcal{M}'', t \in T, d \in \mathcal{D} \tag{3}$$

where $\eta^C_{m''}$ and $\eta^D_{m''}$ represent the charging and discharging efficiencies of storage technology m''. To avoid simultaneous charging and discharging, a binary variable is introduced in the maximum charging and discharging constraints:

$$F_{m'',t,d} \leq S^{\text{chargemax}}_{m''} g_{m'',t,d} \qquad \forall \quad m'' \in \mathcal{M}'', t \in T, d \in \mathcal{D} \tag{4}$$

and

$$P_{m'',t,d} \leq S^{\text{dischargemax}}_{m''}(1 - g_{m'',t,d}) \qquad \forall \quad m'' \in \mathcal{M}'', t \in T, d \in \mathcal{D} \tag{5}$$

Here, $S^{\text{chargemax}}_{m''}$ and $S^{\text{dischargemax}}_{m''}$ denote the charging and discharging capacities, respectively, and $g_{m'',t,d}$ indicates whether the technology is charging ($g_{m'',t,d} = 1$) or discharging ($g_{m'',t,d} = 0$). We assume that the charging and discharging capacities are independent of the storage size. The performance parameters of all storage technologies are presented in Table 3.

Input data

Table S1: Cost parameters of the conventional and alternative chemical processes and technologies. The availability column indicates when the technology becomes available, with 'current layout' indicating that the technology is currently installed at the two sites.

Process	Unit	Available	Size-based CAPEX (λ_m) [k€$_{2022}$/unit]	Size-independent CAPEX (ζ_m) [M€$_{2022}$]	Maintenance costs (ψ) [%]	Lifetime [year]	Source
Conventional cracker	t naphtha/h	current layout	2083	543	4.0	25	1
Conventional cracker with CC	t naphtha/h	short-term	2730	558	4.0	25	1
Electric cracker	t naphtha/h	short-term	2083	543	4.0	25	1
Conventional reformer	MW gas	current layout	574	170	3.8	25	2
Conventional reformer with CC	MW gas	short-term	671	185	3.8	25	2
Electric reformer	MW gas	short-term	525	127	3.9	25	2
AEC	MW electric	short-term	753	-	2.0	25	2
HB process	MW hydrogen	current layout	439	105	3.8	25	2
rWGS	t CO_2/h	short-term	2017	135	2.5	25	3
Methanol synthesis from syngas	t syngas/h	short-term	1969	133	2.5	25	3
Direct methanol synthesis from CO_2	t CO_2/h	mid-term	1613	104	2.5	25	4
MTO	t methanol/h	short-term	1051	66	2.5	25	3
EDH	t ethanol/h	short-term	1162	77	4.0	25	3
PDH	t propane/h	short-term	9676	1034	4.5	25	5
MPW-to-methanol	t MPW/h	short-term	3727	246	4.0	25	6
MPW-to-methanol with CC	t MPW/h	short-term	5360	260	4.0	25	6,7
CO_2 electrolysis	t CO_2/h	mid-term	9461	-	2.0	25	6
ASU	MW electricity	short-term	4224	25	4.2	25	2
Gas-fired boiler	MW gas	short-term	106	-	4.0	25	1
Electric boiler	MW electricity	short-term	154	-	1.0	25	1

Calculation of Weighted Average Product Price

The weighted average product price (WAPP) for the Chemelot and Zeeland clusters, shown in Figures 7 and 11, respectively, is calculated as follows:

$$\text{WAPP} = \frac{\sum_i (\text{price}_i \text{production}_i)}{\sum_i \text{production}_i}, \quad \forall \quad i \in \text{ethylene, propylene, ammonia} \tag{6}$$

This metric represents the average product price weighted by production volumes of the three key chemicals: ethylene, propylene, and ammonia. We use average 2024 market prices.[17–19] Based on the modeled outputs, the resulting WAPP is €879 per tonne of product for Chemelot and €868 per tonne for Zeeland.

Complexity and number of design days

To decrease the computational complexity of the optimization problem, we aggregate the 8760-hour time horizon into a representative set of design days. To identify a suitable number of design days, we perform simulations for the Chemelot cluster in 2030 with a greenfield and a brownfield analysis, systematically varying the number of design days. Figure 2 presents the resulting deviations in cost, installed capacity, and computational effort relative to the solution using 10 design days. After evaluating the trade-off between model fidelity and runtime, we adopt a set of 10 design days for the rest of the analysis.

Results

Tables 5-7 report the installed capacity of the different technologies for the baseline scenarios. Tables 8-12 report the installed capacity of the different technologies for the sensitivity analyses carried out in the manuscript, respectively: (i) cluster location and type, (ii) no CO_2 electrolysis, (iii) lower biomass price, (iv) higher emission from MPW gasification, and

(v) larger import/export capacities.

Table S2: Performance parameters of the conventional and alternative chemical processes and technologies. *The gas for the reformer process, the eSMR, and the gas-fired boiler can be a mix of (bio-)methane and cracker gas.

**Process**	**Inputs**	**Input ratio** ($\beta_{m,n''}$)	**Outputs**	**Output ratio** ($\alpha_{m,n'}$)	**Emission factor** (κ_m) **[t/input]**	**Source**
Conventional cracker	{naphtha, steam, electricity}	[1, 0.082, 0.083]	{ethylene, propylene, crackergas}	[0.303, 0.136, 0.781]	0.327	[1]
Conventional cracker with CC	{naphtha, steam, electricity}	[1, 0.402, 0.107]	{ethylene, propylene, crackergas, CO_2}	[0.303, 0.136, 0.781, 0.295]	0.033	[1]
Electric cracker	{naphtha, electricity}	[1, 1.942]	{ethylene, propylene, crackergas, steam}	[0.303, 0.136, 2.649, 0.050]	-	[1]
Conventional reformer	{gas*, steam}	[1, 0.087]	{hydrogen, nitrogen electricity, CO_2,}	[0.761, 0.106, 0.024, 0.154]	0.067	[8]
Conventional reformer with CC	{gas*, steam}	[1, 0.087]	{hydrogen, nitrogen electricity, CO_2,}	[0.761, 0.106, 0.007, 0.215]	0.007	[8]
Electric reformer	{gas*, electricity}	[1, 0.272]	{hydrogen, CO_2}	[0.927, 0.201]	0.003	[9]
AEC	{electricity}	[1]	{hydrogen, heatlowT, oxygen}	[0.629, 0.163, 0.149]	-	[10]
HB process	{hydrogen, nitrogen, electricity}	[1, 0.139, 0.056]	{ammonia, steam}	[0.168, 0.114]	-	[10]
RWGS	{CO_2, hydrogen}	[1, 4.814]	{syngas}	[0.849]	-	[3]
Methanol synthesis from syngas	{syngas, electricity, steam}	[1, 0.117, 0.572]	{methanol}	[0.823]	-	[3]
Direct methanol synthesis from CO_2	{CO_2, hydrogen, electricity, steam}	[1, 4.566, 0.116, 0.301]	{methanol}	[0.685]	-	[4]
MTO	{methanol, electricity}	[1, 0.049]	{ethylene, propylene}	[0.163, 0.165]	-	[3]
EDH	{ethanol, electricity, steam}	[1, 0.069, 0.313]	{ethylene}	[0.592]	-	[3]
PDH	{propane, electricity, steam}	[1, 0.171, 2.440]	{propylene, hydrogen}	[0.859, 1.357]	-	[5]
MPW-to-methanol	{MPW}	[1]	{methanol}	[1.470]	1.349	[6]
MPW-to-methanol with CC	{MPW, steam, electricity}	[1, 1.294, 0.092]	{methanol, CO_2}	[1.470, 1.214]	0.135	[6,7]
CO_2 electrolysis	{CO_2, electricity}	[1, 11.471]	{ethylene, hydrogen}	[0.602, 0.019]	-	[11]
ASU	{electricity}	[1]	{nitrogen, oxygen}	[4.000, 1.227]	-	[10]
Gas-fired boiler	{gas*}	[1]	{steam}	[0.920]	0.203	[1]
Electric boiler	{electricity}	[1]	{steam}	[0.990]	-	[1]

Table S3: The cost and performance parameters of the energy and material storage technologies. *The storage density of the battery is in MWh/m².

	**Unit**	**Charge efficiency (η^C) [%]**	**Discharge efficiency (η^D) [%]**	**Electricity consumption [MWh/t]**	**Storage density [unit/m^3]**	**Chemelot storage capacity [unit]**	**Zeeland storage capacity [unit]**	**Maximum charge/discharge rate [unit/h]**
Ethylene tank	t ethylene	100	100	-	0.566	181312	114906	250
Propylene tank	t ethylene	100	100	-	0.566	181312	114906	250
Ammonia tank	t ammonia	100	100	-	0.681	218000	138158	250
CO_2 buffer tank	t CO_2	100	100	0.045	1.156	369824	234376	650
Hydrogen tank	MWh	100	100	-	0.417	133565	24324	1500
Li-ion battery	MWh	98	97	-	0.040*	6414	4065	700

		**Cost parameters**			
	**Unit**	**CAPEX (λ_m) [k€$_{2022}$/unit]**	**Maintenance costs (ψ) [%]**	**Lifetime [y]**	**Source**
Ethylene tank	t ethylene	26.2	2	25	[1]
Propylene tank	t ethylene	26.2	2	25	
Ammonia tank	t ammonia	8.74	3	25	[10,12]
CO_2 buffer tank	t CO_2	1.04	6	25	[13,14]
Hydrogen tank	MWh	32.2	1	25	[15]
Li-ion battery	MWh	229	1.5	20	[16]

Table S4: The dynamic operation parameters of the considered technologies.

	**Dynamics type**	**OE [%]**	**RT [h]**	**WSP [%]**
Conventional steam cracker	slow	5	10	-
Conventional steam cracker with CC	slow	5	10	-
Electric steam cracker	medium	60	2.5	5
Conventional reformer	slow	10	10	-
Conventional reformer with CC	slow	10	10	-
Electric reformer	medium	60	2.5	5
AEC	medium	80	<1	5
Haber Bosch process	medium	60	5	0
RWGS	medium	60	5	0
Methanol synthesis from syngas	medium	60	5	0
Direct methanol synthesis from CO_2	medium	60	5	0
MTO	medium	60	5	0
EDH	medium	60	5	0
PDH	medium	60	5	0
MPW-to-methanol	medium	60	5	0
MPW-to-methanol with CC	medium	60	5	0
CO_2 electrolysis	medium	80	<1	0
Gas-fired boiler	fast	100	<1	-
Electric boiler	fast	100	<1	-
Air separation unit	medium	60	5	0

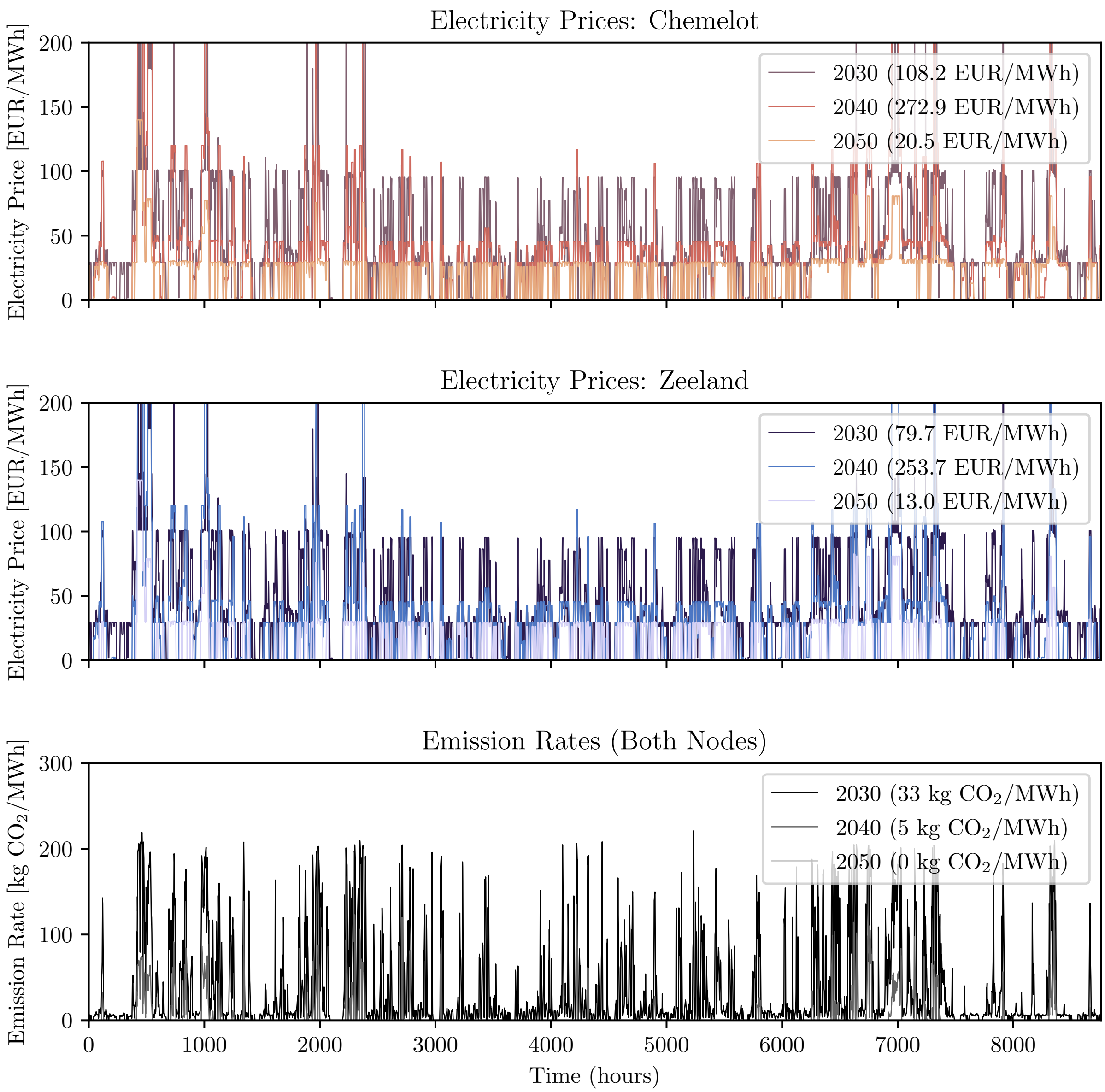

Figure S1: The hourly marginal spot market prices for Chemelot and Zeeland (top two figures) and hourly average specific emissions of the national electricity market (bottom figure) for both clusters for Chemelot in the short-, medium-, and long-term scenarios. The averages are presented between the brackets in the legend.

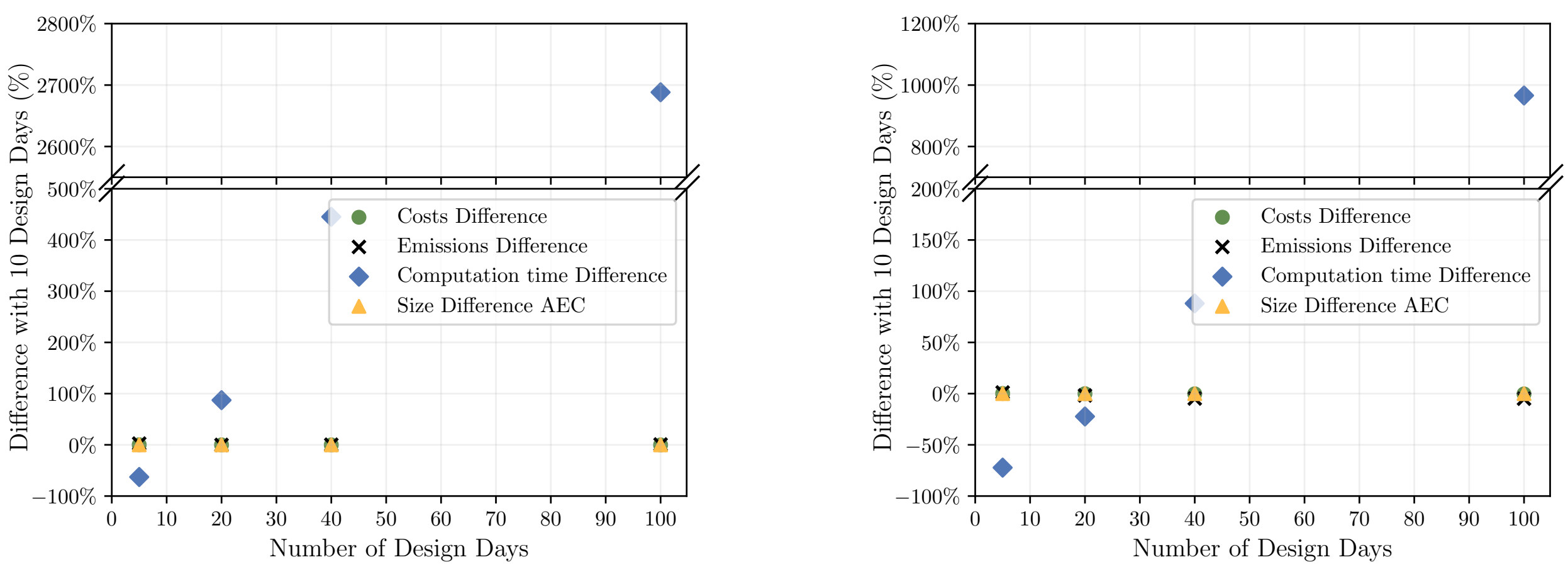

(a) Greenfield analysis (2030)

(b) Brownfield analysis (2030)

Figure S2: The differences in costs, installed capacities, and computation times, compared to the using 10 design days of the Chemelot cluster for a greenfield analysis in subfigure 2a and brownfield analysis in subfigure 2b.

Table S5: Installed capacities for Greenfield, Brownfield and Brownfield with Tight Emission Limit scenarios including Scope 1, 2, and 3 emissions for the short, mid, and long-term interval

Technology	Unit	Greenfield (Scope 1, 2, and 3)			Brownfield (Scope 1, 2, and 3)			Brownfield Tight Emission Limit		
		Short-term	Mid-term	Long-term	Short-term	Mid-term	Long-term	Short-term	Mid-term	Long-term
Conventional cracker	t naphtha/h	0	0	0	-	-	-	-	-	-
Conventional cracker with CC	% captured	0	0	0	-	-	-	-	-	-
Conventional cracker existing	t naphtha/h	-	-	-	0	-	-	0	-	-
Conventional cracker existing with CC	% captured	-	-	-	0	-	-	0	-	-
Electric cracker	t naphtha/h	0	0	0	0	0	0	0	0	0
Conventional reformer	MW gas	0	699	0	-	-	-	-	-	-
Conventional reformer with CC	% captured	0	0	0	-	-	-	-	-	-
Conventional reformer existing	MW gas	-	-	-	0	-	-	0	-	-
Conventional reformer existing with CC	% captured	-	-	-	0	-	-	0	-	-
Electric reformer	MW gas	692	0	733	750	0	0	739	0	0
Electric reformer existing	MW gas	-	-	-	-	750	750	-	739	739
AEC	MW electric	241	463	191	141	134	0	192	75	0
AEC existing	MW electric	-	-	-	-	141	274	-	192	268
HB process	MW hydrogen	840	827	804	0	0	0	0	0	0
HB process existing	MW hydrogen	-	-	-	813	813	813	813	813	813
Methanol synthesis from syngas	t syngas/h	0	0	0	0	0	0	0	0	0
Methanol synthesis from syngas existing	t syngas/h	-	-	-	-	-	-	-	-	-
Direct methanol synthesis from CO_2	t CO_2/h	-	0	0	-	0	0	-	0	0
MTO	t methanol/h	497	486	377	497	0	0	496	0	0
MTO existing	t methanol/h	-	-	-	-	497	497	-	496	496
EDH	t ethanol/h	0	0	0	0	0	0	0	0	0
PDH	t propane/h	64	0	0	64	0	0	64	0	0
PDH existing	t propane/h	-	-	-	-	64	64	-	64	64
MPW-to-methanol	t MPW/h	338	331	257	338	0	0	338	0	0
MPW-to-methanol with CC	% captured	0	34	58	0	0	0	3	0	0
MPW-to-methanol existing	t MPW/h	-	-	-	-	338	338	-	338	338
MPW-to-methanol existing with CC	% captured	-	-	-	-	29	53	-	28	53
CO_2 electrolysis	t CO_2/h	-	106	157	-	111	40	-	111	39
CO_2 electrolysis existing	t CO_2/h	-	-	-	-	-	111	-	-	111
ASU	MW electricity	29	10	28	28	0	0	28	0	0
ASU existing	MW electricity	-	-	-	-	28	28	-	28	28
Gas-fired boiler	MW gas	128	0	0	125	0	0	122	0	0
Gas-fired boiler existing	MW gas	-	-	-	-	125	125	-	122	122
Electric boiler	MW electricity	65	139	124	64	0	51	97	0	20
Electric boiler existing	MW electricity	-	-	-	-	64	64	-	97	97
Ammonia tank	tonne	7225	5629	135	4527	4589	0	4513	4602	0
Ammonia tank existing	tonne	-	-	-	-	4527	9117	-	4513	9116
Li-ion battery	MWh	1212	5963	1	1639	6414	0	1639	6414	0
Li-ion battery existing	MWh	-	-	-	-	1639	8053	-	1639	8053
CO_2 buffer storage	tonne	0	6461	50	0	9813	0	2135	7669	0
CO_2 buffer storage existing	tonne	-	-	-	-	-	9813	-	2135	9805
Hydrogen tank	MWh	162	71	0	166	8342	0	270	8471	0
Hydrogen tank existing	MWh	-	-	-	-	166	8508	-	270	8741
Ethylene tank	tonne	0	7112	49	0	1365	0	0	5336	0
Ethylene tank existing	tonne	-	-	-	-	-	1365	-	-	5336
Propylene tank	tonne	0	571	20	0	4449	0	0	498	0
Propylene tank existing	tonne	-	-	-	-	-	4449	-	-	498

Table S6: Installed capacities for Greenfield and Brownfield scenarios including Scope 1 and 2 emissions for the short, mid, and long-term interval

		Greenfield (Scope 1 and 2)			Brownfield (Scope 1 and 2)		
Technology	Unit	Short-term	Mid-term	Long-term	Short-term	Mid-term	Long-term
Conventional cracker	t naphtha/h	497	307	0	-	-	-
Conventional cracker with CC	% captured	0	87	0	-	-	-
Conventional cracker existing	t naphtha/h	-	-	-	499	0	-
Conventional cracker existing with CC	% captured	-	-	-	0	0	-
Electric cracker	t naphtha/h	0	0	375	0	0	0
Conventional reformer	MW gas	0	1056	0	-	-	-
Conventional reformer with CC	% captured	0	0	0	-	-	-
Conventional reformer existing	MW gas	-	-	-	0	-	-
Conventional reformer existing with CC	% captured	-	-	-	0	-	-
Electric reformer	MW gas	722	0	1032	784	0	617
Electric reformer existing	MW gas	-	-	-	-	784	784
AEC	MW electric	278	0	1277	198	0	895
AEC existing	MW electric	-	-	-	-	198	198
HB process	MW hydrogen	845	806	804	0	0	0
HB process existing	MW hydrogen	-	-	-	813	813	813
Methanol synthesis from syngas	t syngas/h	0	0	193	0	0	262
Direct methanol synthesis from CO_2	t CO_2/h	-	0	0	-	0	0
MTO	t methanol/h	0	0	159	0	37	179
MTO existing	t methanol/h	-	-	-	-	-	37
EDH	t ethanol/h	0	0	0	0	0	0
PDH	t propane/h	0	0	0	0	149	0
PDH existing	t propane/h	-	-	-	-	-	149
MPW-to-methanol	t MPW/h	0	0	0	0	25	0
MPW-to-methanol with CC	% captured	0	0	0	0	83	0
MPW-to-methanol existing	t MPW/h	-	-	-	-	-	25
MPW-to-methanol existing with CC	% captured	-	-	-	-	-	0
CO_2 electrolysis	t CO_2/h	-	152	3	-	139	0
CO_2 electrolysis existing	t CO_2/h	-	-	-	-	-	139
ASU	MW electricity	29	0	28	28	0	0
ASU existing	MW electricity	-	-	-	-	28	28
Gas-fired boiler	MW gas	2	28	0	0	334	0
Gas-fired boiler existing	MW gas	-	-	-	-	-	334
Electric boiler	MW electricity	0	123	0	0	1	425
Electric boiler existing	MW electricity	-	-	-	-	-	1
Ammonia tank	tonne	8111	1395	0	4483	4414	0
Ammonia tank existing	tonne	-	-	-	-	4483	8897
Li-ion battery	MWh	1337	4798	0	1724	6408	0
Li-ion battery existing	MWh	-	-	-	-	1724	8133
CO_2 buffer storage	tonne	0	1056	0	0	2110	0
CO_2 buffer storage existing	tonne	-	-	-	-	-	2110
Hydrogen tank	MWh	129	1	0	238	411	0
Hydrogen tank existing	MWh	-	-	-	-	238	649
Ethylene tank	tonne	0	3307	0	9	497	0
Ethylene tank existing	tonne	-	-	-	-	9	506
Propylene tank	tonne	0	5691	0	9	9219	0
Propylene tank existing	tonne	-	-	-	-	9	9227

Table S7: Maximum import and export capacities for the Greenfield and Brownfield scenarios, including and excluding scope 3 emissions, for the short, mid, and long-term intervals

			Greenfield			Brownfield	
Carrier	Unit	Short-term	Mid-term	Long-term	Short-term	Mid-term	Long-term
**scope 1, 2, and 3**							
Electricity grid import	MW (% of max)	750 (100%)	2000 (100%)	2500 (100%)	750 (100%)	2000 (100%)	2500 (100%)
CO_2 T&S	t CO_2/h (% of max)	95 (83%)	114 (100%)	148 (100%)	107 (94%)	114 (100%)	148 (100%)
MPW import	t MPW/h (% of max)	338 (100%)	331 (87%)	257 (60%)	338 (100%)	338 (89%)	338 (80%)
Methane import	MW gas	803	706	740	852	757	757
Bio-methane import	MW gas	0	0	0	0	0	0
DAC-CO_2 import	t CO_2/h	0	0	0	0	0	0
Bio-propane import	MW	64	0	0	64	0	0
**Tight Emission Limit (scope 1, 2, and 3)**		-	-	-			
Electricity grid import	MW (% of max)	-	-	-	750 (100%)	2000 (100%)	2500 (100%)
CO_2 T&S	t CO_2/h (% of max)	-	-	-	114 (100%)	114 (100%)	148 (100%)
MPW import	t MPW/h (% of max)	-	-	-	338 (100%)	338 (89%)	338 (80%)
Methane import	MW gas	-	-	-	845	747	747
Bio-methane import	MW gas	-	-	-	0	0	0
DAC-CO_2 import	t CO_2/h	-	-	-	0	0	0
Bio-propane import	MW	-	-	-	64	0	0
**scope 1 and 2**							
Electricity grid import	MW (% of max)	750 (100%)	2000 (100%)	2500 (100%)	750 (100%)	2000 (100%)	2500 (100%)
CO_2 T&S	t CO_2/h (% of max)	101 (88%)	114 (100%)	0 (0%)	114 (100%)	114 (100%)	0 (0%)
MPW import	t MPW/h (% of max)	0 (0%)	0 (0%)	0 (0%)	0 (0%)	25 (7%)	0 (0%)
Methane import	MW gas	341	850	746	421	1112	1408
Bio-methane import	MW gas	0	0	0	0	0	0
DAC-CO_2 import	t CO_2/h	0	0	47	0	0	78
Bio-propane import	MW	0	0	0	0	149	149

Table S8: Installed capacities for sensitivity case: Zeeland

Technology	Unit	Greenfield (Scope 1, 2, and 3)			Brownfield (Scope 1, 2, and 3)		
		Short-term	Mid-term	Long-term	Short-term	Mid-term	Long-term
Conventional cracker	t naphtha/h	0	0	0	-	-	-
Conventional cracker with CC	% captured	0	0	0	-	-	-
Conventional cracker existing	t naphtha/h	-	-	-	0	-	-
Conventional cracker existing with CC	% captured	-	-	-	0	-	-
Electric cracker	t naphtha/h	0	0	0	0	0	0
Conventional reformer	MW gas	0	1627	0	-	-	-
Conventional reformer with CC	% captured	0	0	0	-	-	-
Conventional reformer existing	MW gas	-	-	-	0	-	-
Conventional reformer existing with CC	% captured	-	-	-	0	-	-
Electric reformer	MW gas	750	0	951	895	0	0
Electric reformer existing	MW gas	-	-	-	-	895	895
AEC	MW electric	952	0	556	895	0	0
AEC existing	MW electric	-	-	-	-	895	895
HB process	MW hydrogen	1325	1242	1238	0	43	0
HB process existing	MW hydrogen	-	-	-	1248	1248	1291
Methanol synthesis from syngas	t syngas/h	0	0	0	0	0	0
Direct methanol synthesis from CO_2	t CO_2/h	-	0	0	-	0	0
MTO	t methanol/h	689	575	440	689	0	0
MTO existing	t methanol/h	-	-	-	-	689	689
EDH	t ethanol/h	0	0	0	0	0	0
PDH	t propane/h	88	0	0	88	0	0
PDH existing	t propane/h	-	-	-	-	88	88
MPW-to-methanol	t MPW/h	469	391	299	469	0	0
MPW-to-methanol with CC	% captured	0	58	89	0	0	0
MPW-to-methanol existing	t MPW/h	-	-	-	-	469	469
MPW-to-methanol existing with CC	% captured	-	-	-	-	27	81
CO_2 electrolysis	t CO_2/h	-	212	262	-	146	108
CO_2 electrolysis existing	t CO_2/h	-	-	-	-	-	146
ASU	MW electricity	46	0	43	43	1	0
ASU existing	MW electricity	-	-	-	-	43	45
Gas-fired boiler	MW gas	501	333	0	503	0	0
Gas-fired boiler existing	MW gas	-	-	-	-	503	503
Electric boiler	MW electricity	379	675	558	383	7	156
Electric boiler existing	MW electricity	-	-	-	-	383	390
Ammonia tank	tonne	16566	2325	0	5113	10715	0
Ammonia tank existing	tonne	-	-	-	-	5113	15828
Li-ion battery	MWh	1476	3578	0	1581	4065	0
Li-ion battery existing	MWh	-	-	-	-	1581	5646
CO_2 buffer storage	tonne	225	54	0	182	1332	0
CO_2 buffer storage existing	tonne	-	-	-	-	182	1514
Hydrogen tank	MWh	958	0	0	1715	13841	0
Hydrogen tank existing	MWh	-	-	-	-	1715	15556
Ethylene tank	tonne	0	5336	0	0	5725	0
Ethylene tank existing	tonne	-	-	-	-	-	5725
Propylene tank	tonne	0	10252	0	0	3790	0
Propylene tank existing	tonne	-	-	-	-	-	3790

Table S9: Installed capacities for sensitivity case: No CO_2 electrolyzers

Technology	Unit	Greenfield (Scope 1, 2, and 3)			Brownfield (Scope 1, 2, and 3)		
		Short-term	Mid-term	Long-term	Short-term	Mid-term	Long-term
Conventional cracker	t naphtha/h	0	-	-	-	-	-
Conventional cracker with CC	% captured	0	-	-	-	-	-
Conventional cracker existing	t naphtha/h	-	-	-	0	-	-
Conventional cracker existing with CC	% captured	-	-	-	0	-	-
Electric cracker	t naphtha/h	0	0	0	0	0	0
Conventional reformer	MW gas	0	-	-	-	-	-
Conventional reformer with CC	% captured	0	-	-	-	-	-
Conventional reformer existing	MW gas	-	-	-	0	-	-
Conventional reformer existing with CC	% captured	-	-	-	0	-	-
Electric reformer	MW gas	696	0	0	752	0	0
Electric reformer existing	MW gas	-	-	-	-	752	752
AEC	MW electric	237	1330	2160	148	623	466
AEC existing	MW electric	-	-	-	-	148	770
HB process	MW hydrogen	844	850	804	0	0	0
HB process existing	MW hydrogen	-	-	-	813	813	813
Methanol synthesis from syngas	t syngas/h	0	0	0	0	0	56
Direct methanol synthesis from CO_2	t CO_2/h	-	0	122	-	0	0
MTO	t methanol/h	497	560	665	497	0	46
MTO existing	t methanol/h	-	-	-	-	497	497
EDH	t ethanol/h	0	0	0	0	0	0
PDH	t propane/h	64	40	0	64	0	0
PDH existing	t propane/h	-	-	-	-	64	64
MPW-to-methanol	t MPW/h	338	381	395	338	0	0
MPW-to-methanol with CC	% captured	0	10	31	0	0	0
MPW-to-methanol existing	t MPW/h	-	-	-	-	338	338
MPW-to-methanol existing with CC	% captured	-	-	-	-	8	22
CO_2 electrolysis	t CO_2/h	-	-	-	-	-	-
CO_2 electrolysis existing	t CO_2/h	-	-	-	-	-	-
ASU	MW electricity	29	30	28	28	0	0
ASU existing	MW electricity	-	-	-	-	28	28
Gas-fired boiler	MW gas	128	64	0	124	5	0
Gas-fired boiler existing	MW gas	-	-	-	-	124	130
Electric boiler	MW electricity	65	92	124	64	91	57
Electric boiler existing	MW electricity	-	-	-	-	64	156
Ammonia tank	tonne	7908	11258	0	4210	4907	0
Ammonia tank existing	tonne	-	-	-	-	4210	9117
Li-ion battery	MWh	1220	6414	0	1663	6414	0
Li-ion battery existing	MWh	-	-	-	-	1663	8077
CO_2 buffer storage	tonne	0	5221	0	0	0	0
CO_2 buffer storage existing	tonne	-	-	-	-	-	-
Hydrogen tank	MWh	129	32493	0	195	3234	0
Hydrogen tank existing	MWh	-	-	-	-	195	3429
Ethylene tank	tonne	0	0	0	0	0	81
Ethylene tank existing	tonne	-	-	-	-	-	-
Propylene tank	tonne	0	0	0	0	0	79
Propylene tank existing	tonne	-	-	-	-	-	-

Table S10: Installed capacities for sensitivity case: Optimistic Bio-Feedstock Prices

Technology	Unit	Greenfield (Scope 1, 2, and 3)			Brownfield (Scope 1, 2, and 3)		
		Short-term	Mid-term	Long-term	Short-term	Mid-term	Long-term
Conventional cracker	t naphtha/h	0	0	0	-	-	-
Conventional cracker with CC	% captured	0	0	0	-	-	-
Conventional cracker existing	t naphtha/h	-	-	-	0	-	-
Conventional cracker existing with CC	% captured	-	-	-	0	-	-
Electric cracker	t naphtha/h	0	0	0	0	0	0
Conventional reformer	MW gas	0	895	0	-	-	-
Conventional reformer with CC	% captured	0	77	0	-	-	-
Conventional reformer existing	MW gas	-	-	-	0	-	-
Conventional reformer existing with CC	% captured	-	-	-	0	-	-
Electric reformer	MW gas	759	0	731	744	0	0
Electric reformer existing	MW gas	-	-	-	-	744	744
AEC	MW electric	2	0	0	0	21	0
AEC existing	MW electric	-	-	-	-	-	21
HB process	MW hydrogen	828	806	804	0	0	0
HB process existing	MW hydrogen	-	-	-	813	813	813
Methanol synthesis from syngas	t syngas/h	0	0	0	0	0	0
Direct methanol synthesis from CO_2	t CO_2/h	-	0	0	-	0	0
MTO	t methanol/h	253	0	0	253	0	0
MTO existing	t methanol/h	-	-	-	-	253	253
EDH	t ethanol/h	0	0	0	0	0	0
PDH	t propane/h	157	157	157	157	0	0
PDH existing	t propane/h	-	-	-	-	157	157
MPW-to-methanol	t MPW/h	172	0	0	172	0	0
MPW-to-methanol with CC	% captured	0	0	0	0	0	0
MPW-to-methanol existing	t MPW/h	-	-	-	-	172	172
MPW-to-methanol existing with CC	% captured	-	-	-	-	0	0
CO_2 electrolysis	t CO_2/h	-	145	138	-	103	39
CO_2 electrolysis existing	t CO_2/h	-	-	-	-	-	103
ASU	MW electricity	29	4	28	28	0	0
ASU existing	MW electricity	-	-	-	-	28	28
Gas-fired boiler	MW gas	369	402	0	367	0	0
Gas-fired boiler existing	MW gas	-	-	-	-	367	367
Electric boiler	MW electricity	292	246	294	293	0	0
Electric boiler existing	MW electricity	-	-	-	-	293	293
Ammonia tank	tonne	4738	1189	0	3657	5433	0
Ammonia tank existing	tonne	-	-	-	-	3657	9090
Li-ion battery	MWh	1240	5424	0	1433	6414	0
Li-ion battery existing	MWh	-	-	-	-	1433	7847
CO_2 buffer storage	tonne	0	1888	0	0	118	150
CO_2 buffer storage existing	tonne	-	-	-	-	-	118
Hydrogen tank	MWh	69	0	0	0	695	0
Hydrogen tank existing	MWh	-	-	-	-	-	695
Ethylene tank	tonne	0	7733	0	0	477	0
Ethylene tank existing	tonne	-	-	-	-	-	477
Propylene tank	tonne	0	985	0	0	7148	0
Propylene tank existing	tonne	-	-	-	-	-	7148

Table S11: Installed capacities for sensitivity case: Direct Emissions from MPW Gasification

Technology	Unit	Greenfield (Scope 1, 2, and 3)			Brownfield (Scope 1, 2, and 3)		
		Short-term	Mid-term	Long-term	Short-term	Mid-term	Long-term
Conventional cracker	t naphtha/h	0	0	0	-	-	-
Conventional cracker with CC	% captured	0	0	0	-	-	-
Conventional cracker existing	t naphtha/h	-	-	-	0	-	-
Conventional cracker existing with CC	% captured	-	-	-	0	-	-
Electric cracker	t naphtha/h	0	0	0	0	0	0
Conventional reformer	MW gas	0	896	0	-	-	-
Conventional reformer with CC	% captured	0	81	0	-	-	-
Conventional reformer existing	MW gas	-	-	-	0	-	-
Conventional reformer existing with CC	% captured	-	-	-	0	-	-
Electric reformer	MW gas	760	0	745	744	0	0
Electric reformer existing	MW gas	-	-	-	-	744	744
AEC	MW electric	0	0	0	0	0	6
AEC existing	MW electric	-	-	-	-	-	-
HB process	MW hydrogen	828	807	804	0	0	0
HB process existing	MW hydrogen	-	-	-	813	813	813
Methanol synthesis from syngas	t syngas/h	0	0	0	0	0	0
Direct methanol synthesis from CO_2	t CO_2/h	-	0	0	-	0	0
MTO	t methanol/h	253	0	0	253	27	0
MTO existing	t methanol/h	-	-	-	-	253	280
EDH	t ethanol/h	0	0	0	0	0	0
PDH	t propane/h	157	157	140	157	0	0
PDH existing	t propane/h	-	-	-	-	157	157
MPW-to-methanol	t MPW/h	172	0	0	172	18	0
MPW-to-methanol with CC	% captured	0	0	0	0	87	0
MPW-to-methanol existing	t MPW/h	-	-	-	-	172	191
MPW-to-methanol existing with CC	% captured	-	-	-	-	0	0
CO_2 electrolysis	t CO_2/h	-	146	163	-	129	32
CO_2 electrolysis existing	t CO_2/h	-	-	-	-	-	129
ASU	MW electricity	29	4	28	28	0	0
ASU existing	MW electricity	-	-	-	-	28	28
Gas-fired boiler	MW gas	368	401	0	356	11	0
Gas-fired boiler existing	MW gas	-	-	-	-	356	367
Electric boiler	MW electricity	294	195	252	293	0	0
Electric boiler existing	MW electricity	-	-	-	-	293	293
Ammonia tank	tonne	4686	1661	0	3358	5390	0
Ammonia tank existing	tonne	-	-	-	-	3358	8748
Li-ion battery	MWh	1240	4958	0	1470	6414	0
Li-ion battery existing	MWh	-	-	-	-	1470	7884
CO_2 buffer storage	tonne	0	1811	0	0	1619	0
CO_2 buffer storage existing	tonne	-	-	-	-	-	1619
Hydrogen tank	MWh	70	2	0	0	495	0
Hydrogen tank existing	MWh	-	-	-	-	-	495
Ethylene tank	tonne	0	11419	0	0	1134	0
Ethylene tank existing	tonne	-	-	-	-	-	1134
Propylene tank	tonne	0	500	0	0	6809	0
Propylene tank existing	tonne	-	-	-	-	-	6809

Table S12: Maximum import and export capacities for all sensitivity cases

Carrier	Unit	Greenfield (Scope 1, 2, and 3)			Brownfield (Scope 1, 2, and 3)		
		Short-term	Mid-term	Long-term	Short-term	Mid-term	Long-term
**Zeeland**							
Electricity grid import	MW (% of max)	1900 (100%)	3250 (100%)	4600 (100%)	1900 (100%)	3250 (100%)	4600 (100%)
CO_2 T&S	t CO_2/h (% of max)	273 (96%)	354 (100%)	190 (26%)	260 (91%)	243 (69%)	407 (55%)
MPW import	t MPW/h (% of max)	469 (100%)	391 (74%)	299 (51%)	469 (100%)	469 (89%)	469 (80%)
Methane import	MW gas	1252	1960	951	1350	1211	895
Bio-methane import	MW gas	0	0	0	0	0	0
DAC-CO_2 import	t CO_2/h	0	0	0	0	0	0
Bio-propane import	MW	88	0	0	88	0	0
**No CO_2 electrolyzers**							
Electricity grid import	MW (% of max)	750 (100%)	2000 (100%)	2500 (100%)	750 (100%)	2000 (100%)	2442 (98%)
CO_2 T&S	t CO_2/h (% of max)	96 (84%)	62 (54%)	1 (1%)	107 (94%)	114 (100%)	83 (56%)
MPW import	t MPW/h (% of max)	338 (100%)	381 (100%)	395 (93%)	338 (100%)	338	338
Methane import	MW gas	803	71	7	854	828	759
Bio-methane import	MW gas	0	0	0	0	0	0
DAC-CO_2 import	t CO_2/h	0	0	0	0	0	0
Bio-propane import	MW	64	40	0	64	64	64
**Optimistic Bio-Feedstock Prices**							
Electricity grid import	MW (% of max)	750 (100%)	2000 (100%)	2259 (90%)	750 (100%)	2000 (100%)	2500 (100%)
CO_2 T&S	t CO_2/h (% of max)	109 (96%)	94 (82%)	0 (0%)	106 (93%)	108 (95%)	0 (0%)
MPW import	t MPW/h (% of max)	172 (51%)	0 (0%)	0 (0%)	172 (51%)	172 (45%)	0 (0%)
Methane import	MW gas	1083	0	0	1067	0	0
Bio-methane import	MW gas	0	1304	738	0	1067	1068
DAC-CO_2 import	t CO_2/h	0	0	35	0	0	76
Bio-propane import	MW	157	157	157	157	157	157
**Direct Emissions from MPW Gasification**							
Electricity grid import	MW (% of max)	750 (100%)	2000 (100%)	2500 (100%)	750 (100%)	2000 (100%)	2500 (100%)
CO_2 T&S	t CO_2/h (% of max)	109 (96%)	94 (82%)	0 (0%)	106 (93%)	46 (40%)	106 (71%)
MPW import	t MPW/h (% of max)	172 (51%)	0 (0%)	0 (0%)	172 (51%)	18 (5%)	0 (0%)
Methane import	MW gas	1089	1305	0	1067	1093	162
Bio-methane import	MW gas	0	0	752	0	0	751
DAC-CO_2 import	t CO_2/h	0	0	57	0	0	57
Bio-propane import	MW	157	157	140	157	157	157

References

(1) Tiggeloven, J. L.; Faaij, A. P.; Kramer, G. J.; Gazzani, M. Optimization of Electric Ethylene Production: Exploring the Role of Cracker Flexibility, Batteries, and Renewable Energy Integration. *Industrial and Engineering Chemistry Research* **2023**, *62*, 16360–16382.

(2) Tiggeloven, J. L.; Faaij, A. P.; Kramer, G. J.; Gazzani, M. Optimizing Emissions Reduction in Ammonia-Ethylene Chemical Clusters: Synergistic Integration of Electrification, Carbon Capture, and Hydrogen. *Industrial and Engineering Chemistry Research* **2025**, *64*, 4479–4497.

(3) Ding, S.; Kramer, G. J.; Faaij, A.; Gazzani, M. Manuscript submitted for publication.

(4) Allgoewer, L.; Becattini, V.; Patt, A.; Grandjean, P.; Wiegner, J. F.; Gazzani, M.; Moretti, C. Cost-Effective Locations for Producing Fuels and Chemicals from Carbon Dioxide and Low-Carbon Hydrogen in the Future. *Ind. Eng. Chem. Res.* **2024**, *63*, 13660–13676.

(5) Brencio, C. Membrane-assisted direct dehydrogenation technology for light olefins production: Process design and experimental evaluation. Ph.D. thesis, Eindhoven University of Technology, 2023.

(6) Afzal, S.; Singh, A.; Nicholson, S. R.; Uekert, T.; DesVeaux, J. S.; Tan, E. C.; Dutta, A.; Carpenter, A. C.; Baldwin, R. M.; Beckham, G. T. Techno-economic analysis and life cycle assessment of mixed plastic waste gasification for production of methanol and hydrogen. *Green Chem.* **2023**, *25*, 5068–5085.

(7) Weimann, L.; Dubbink, G.; van der Ham, L.; Gazzani, M. A thermodynamic-based mixed-integer linear model of post-combustion carbon capture for reliable use in energy system optimisation. *Appl. Energy* **2023**, *336*, 120738.

(8) IEAGHG Techno-Economic Evaluation of HYCO Plant integrated to Ammonia / Urea or Methanol Production with CCS. 2017.

(9) IEAGHG Low-Carbon Hydrogen from Natural Gas: Global Roadmap. 2022.

(10) DEA Technology Data for Renewable Fuels. 2017; http://www.ens.dk/teknologikatalog, Accessed 2024-03-19.

(11) Sisler, J.; Khan, S.; Ip, A. H.; Schreiber, M. W.; Jaffer, S. A.; Bobicki, E. R.; Dinh, C. T.; Sargent, E. H. Ethylene Electrosynthesis: A Comparative Techno-economic Analysis of Alkaline vs Membrane Electrode Assembly vs CO_2-CO-C_2H_4 Tandems. *ACS Energy Lett.* **2021**, *6*, 997–1002.

(12) Palys, M. J.; Kuznetsov, A.; Tallaksen, J.; Reese, M.; Daoutidis, P. A novel system for ammonia-based sustainable energy and agriculture: Concept and design optimization. *Chem. Eng. Process. Process Intensif.* **2019**, *140*, 11–21.

(13) Roussanaly, S.; Deng, H.; Skaugen, G.; Gundersen, T. At what pressure shall CO_2 be transported by ship? An indepth cost comparison of 7 and 15 barg shipping. *Energies* **2021**, *14*.

(14) Gardarsdottir, S. O.; De Lena, E.; Romano, M.; Roussanaly, S.; Voldsund, M.; Pérez-Calvo, J.-F.; Berstad, D.; Fu, C.; Anantharaman, R.; Sutter, D.; others Comparison of technologies for CO2 capture from cement productionâPart 2: Cost analysis. *Energies* **2019**, *12*, 542.

(15) DEA Technology Data-Energy storage. 2022; http://www.ens.dk/teknologikatalog, Accessed 2024-03-14.

(16) IRENA Electricity storage and renewables: costs and markets to 2030. 2017; https://www.irena.org/publications/2017/Oct/Electricity-storage-and-renewables-costs-and-markets.

(17) Business Analytiq Ethylene Price Index. 2024; https://businessanalytiq.com/procurementanalytics/index/ethylene-price-index/, Accessed 2025-04-28.

(18) Business Analytiq Propylene Price Index. 2024; https://businessanalytiq.com/procurementanalytics/index/propylene-price-index/, Accessed 2025-04-28.

(19) Business Analytiq Ammonia Price Index. 2024; https://businessanalytiq.com/procurementanalytics/index/ammonia-price-index/, Accessed 2025-04-28.
